# Impact of oral conditions on oral health-related quality of life among Indians- a systematic review and Meta-analysis

**DOI:** 10.1186/s12955-023-02170-6

**Published:** 2023-08-31

**Authors:** Anju James, Chandrashekar Janakiram, R. V. Meghana, Vijay S. Kumar, Anitha R. Sagarkar, Yuvraj B. Y.

**Affiliations:** 1 Department of Public Health Dentistry, Amrita School of Dentistry,, Amrita Vishwa Vidyapeetham, Edapally, Ernakulum, 682041 India; 2 Amrita Centre for Evidence Based Oral Health, Amrita School of Dentistry, Ernakulum, 682041 India; 3 Department of Public Health Dentistry, Faculty of Dental Sciences, Ramaiah University of Applied Sciences, Bangalore, Karnataka India; 4 Department of Public Health, Rajiv Gandhi University of Health Sciences, Bangalore, India

**Keywords:** Indian population, Oral conditions, Oral Health-Related Quality of Life, Dental caries, Periodontal disease

## Abstract

**Objective:**

This review assessed the impact of oral conditions on Oral Health Related Quality of Life among Indians.

**Methods:**

Databases, including PubMed and Scopus, CINAHL, Web of Science, PsycInfo were systematically searched for English Language studies conducted among Indians up to July 2022. Two independent reviewers assessed studies selected for retrieval for methodological quality using standardised quality assessment instruments for analytical cross-sectional studies in JBI SUMARI.

**Results:**

Fourty one publications were included in this review (*N* = 23,090). Studies includes both cross sectional study and Randomized Controlled Trials. Based on the JBI critical appraisal tools, the quality of the included studies was low to high. Twenty-six studies were considered for the meta-analysis. Individuals with dental caries [OR: 3.54 (95% CI 2.24- 5.60), ten studies, 4945 participants] and malocclusion [ OR: 5.44 (95% CI 1.61, 18.39), six studies, 3720 participants] had poor OHRQoL compared to individuals without oral conditions.

**Conclusions:**

Despite the various definitions of the exposures and instruments used to assess Oral Health-Related Quality of Life, our review found that people with dental caries and malocclusion have a significantly higher experience of poor quality of life.

**Prospero Systematic Review Registration No.:**

CRD42021277874.

**Supplementary Information:**

The online version contains supplementary material available at 10.1186/s12955-023-02170-6.

## Introduction

Oral health is a crucial indicator of general health, well-being, and Quality of life [1]. The World Health Organization (WHO) associates a "person's capacity to bite, chew, smile, and speak with their psychosocial status when defining *oral health* as a disorder-free state" [2]. Oral health conditions affect 3.5 billion people worldwide, according to the Global Burden of Disease Study 2017 [3]. Oral conditions are becoming more common in most lower- and middle-income countries as a result of increased urbanisation and lifestyle changes [4].

In India, the frequency of dental caries is 49%, 60%, and 84% among young children, adults, and the elderly [5]. Nearly half of the Indian population experiences levels of periodontal disease [6]. Complete tooth mortality is 10.7%, and partial tooth mobility is 58.8% [7]. The prevalence of malocclusion among children aged 8 to 15 is 35.40% [8]), and 15 states in *India* are endemic to *fluorosis*. Oral cancer is the most common in India, accounting for one-third of the global burden [9]. Oral conditions are linked with low Oral Health-Related Quality of Life (OHRQoL) [10].

"Oral Health-Related Quality of Life is integral to general health and well-being" [11]. 'Oral health status' nomenclature is now considered 'Oral Health-Related Quality of Life [12].

Quality of life (QoL) refers to a person's position in life "within the context of the culture and value systems in which they live and for their objectives, expectations, standards, and concerns" [13]. Quality of Life is a valid criterion for evaluating patients in many physical and mental healthcare areas, including dental health. Cohen and Jago [14] were among the first to advocate for the development of socio-dental indicators, as studies showed that oral conditions and their consequences interact with social life. The subjective assessment of OHRQoL "reflects people's comfort while eating, sleeping, and participating in social interaction; their sense of self-worth; and their satisfaction with their oral health" [15]. The OHRQoL is the outcome of a complex interaction between and among oral health problems, social and contextual attributes [16], and the rest of one's body [17].

Positive and negative perceptions of oral health and health outcomes have been incorporated into HRQoL and OHRQoL due to the growing emphasis on health policy that addresses health promotion and illness prevention [18]. The various tools available to assess the OHRQoL include the Early Childhood Oral Health Impact Scale (ECOHIS) [19], Child Oral Impact on Daily Performances (Child-OIDP) [20], Scale of Oral Health Outcomes (SOHO) [21], The Child Perceptions Questionnaire for children aged 8 to 10 years [22], The Child Perceptions Questionnaire for children aged 11 to 14 years (CPQ11-14) [23], Oral Health Impact Profile for 14 items (OHIP-14) [24], Oral Impacts on Daily Performances (OIDP) [25], Dental Impact on Daily Living (DIDL) [26], Geriatric Oral Health Assessment Index (GOHAI) [27], Dental Impact profile (DIP) [28], Social Impact on Dental Disease (SIDD) [28].

When identifying suitable treatment goals and outcomes, OHRQoL assessment allows a shift away from conventional medical/dental criteria and toward evaluation and care that focuses on a people's psychological and social experience as well as physical functionality [29]. Understanding the impact of oral problems on OHRQoL is crucial for the public health system, research, and decision-making on methods for improving and preventing oral health [2]. OHRQoL has been deemed a health priority by the U.S. Surgeon General [15], and "QoL concerns are now at the forefront of public health policy" [12].

Few systematic reviews have examined the effects of certain oral conditions on OHRQoL, Early Childhood Caries [30], Periodontal disease [31], Traumatic Dental Injuries (TDI) [32], and malocclusion [33]. An earlier review assessed impacts of oral disease on OHRQoL irrespective of geographical locations, with less representative Indian studies [34] and among the Latin American and Caribbean populations [2]. As OHRQoL is based on the social and cultural context [12] this review will helps in assessing impact of oral conditions on OHRQoL among Indians.

Over the last decade, several studies have assessed certain oral conditions affecting OHRQoL among the Indian population showing inconclusive impact. Therefore, a preliminary search was conducted using PROSPERO, MEDLINE, the Cochrane Database of Systematic Reviews, and the JBI Database of Systematic Reviews. This search revealed no systematic reviews with meta-analyses currently in progress or published on the effect of oral conditions on OHRQoL among Indians. Since OHRQoL is a subjective perception based on the social context, the evidence of oral conditions' impact is vital for health policy and programs. Patients' subjective evaluations of the healthcare decision-making process are changing the dynamics of healthcare delivery, current health monitoring, and research [35]. As a result, this study aimed to perform a systematic review of studies conducted in India to determine how oral conditions affected OHRQoL.

## Review question

What is the Impact of oral conditions (E) on oral health and quality of life (O) when compared to individuals without oral conditions (C) among Indians (P)?

## Methodology

For the systematic review report, the preferred reporting items for systematic reviews and meta-analysis (PRISMA) guidelines [36] were followed and registered in the prospective international register of systematic reviews (PROSPERO) under the registration number CRD42021277874*.*

Cross-sectional studies and Randomised Controlled Trials that addressed associations between oral conditions (Dental caries, Gingivitis, Periodontal Disease, Malocclusion, Dental Fluorosis, Tooth Loss, and Prosthetic Need) and OHRQoL were included.

The outcome was the OHRQoL assessed by instruments such as CPQ 11-14 [23], ECOHIS [19], FIS (Family Impact Scale) [37], GOHAI [27], OHIA (Oral Health Impact in Adolescents) [38], OHIP-14 (Oral Health Impact Profile) [24], OIDP [25] and WHOQoL (World Health Organization Quality of Life) [39].

### Literature search strategy

For the literature search, a three-step search strategy was used. An initial MEDLINE search was conducted using the keywords "Oral disease," "Quality of Life," and "Indian population." After combining keywords and synonyms with the Boolean terms "AND" and "OR," a search string was created. Second, text words from the title, abstract, and index terms of the identified studies were used to inform the development of a search strategy tailored to each information source. PubMed, Scopus, CINAHL, Web of Science, and PsycInfo were among the databases searched for published studies. To broaden the search, Google Scholar was also used. Supplementary material (S1) shows the search strategy of different databases. The third step involved reviewing the reference lists of all study chosen for critical appraisal in order to find additional information.

### Study selection

Following the electronic search, all citations found were compiled and uploaded to Covidence, and duplicates were removed. Both titles and abstracts were evaluated by two investigators (A.J. and R.V.M.). In the event of ambiguity, the full text was read for a joint decision. The full texts of the abstracts that were screened were obtained and evaluated for eligibility. Any disagreement about whether a study should be included was discussed between the two reviewers until a mutual agreement was reached or a third reviewer (C.J.) was approached. Supplementary material (S2) shows the studies ineligible following full text review.

### Assessment of the methodological quality

After the ineligible studies were excluded, the quality of the eligible studies was assessed by two independent reviewers (A.R.S. and S.V.K) using standardised critical appraisal instruments for analytical cross-sectional studies in JBI SUMARI. The same checklist was used for experimental studies to assess how baseline data was collected and analysed, as that was the desired outcome [40]. In the event of disagreement, a third reviewer's (C.J.) opinion was sought for further discussion. There were eight questions, with answers ranging from "yes," "no," "unclear," and "not applicable." Each study received an overall score based on several "Yes" responses ranging from 0 to 8. Finally, studies were classified based on their score: 0–3, low quality; 4–6, medium quality; and 7–8, high quality [36].

### Data extraction

Data from the included studies were extracted using the customised tool. Each study's data was extracted by two independent reviewers (A.J. and R.V.M). The extracted data included specific details about the study's characteristics, population characteristics, and outcome measures. To ensure consistency during the extraction process, the two independent reviewers met and compared the extracted data from each included study in a Microsoft Word document. No studies necessitated additional information from the corresponding authors.

### Data synthesis

Studies, where possible, were pooled in a statistical meta-analysis using JBI SUMARI software. Data were presented as either odds ratios (for binary outcome) and weighted (or standardized) mean differences (for continuous measures) and their 95% confidence intervals. The standard χ2, Tau2, and I^2^ tests were used to assess heterogeneity. To estimate the pooled effect, the random-effects model with heterogeneity taken from an inverse variance model was used. Subgroup analyses were conducted for dental caries based on the tool to assess the OHRQoL. When statistical pooling was impossible, or when there is less than four studies to pool the data the findings were presented in descriptive form.

### Assessing the certainty of the findings

**Fig. 1:**
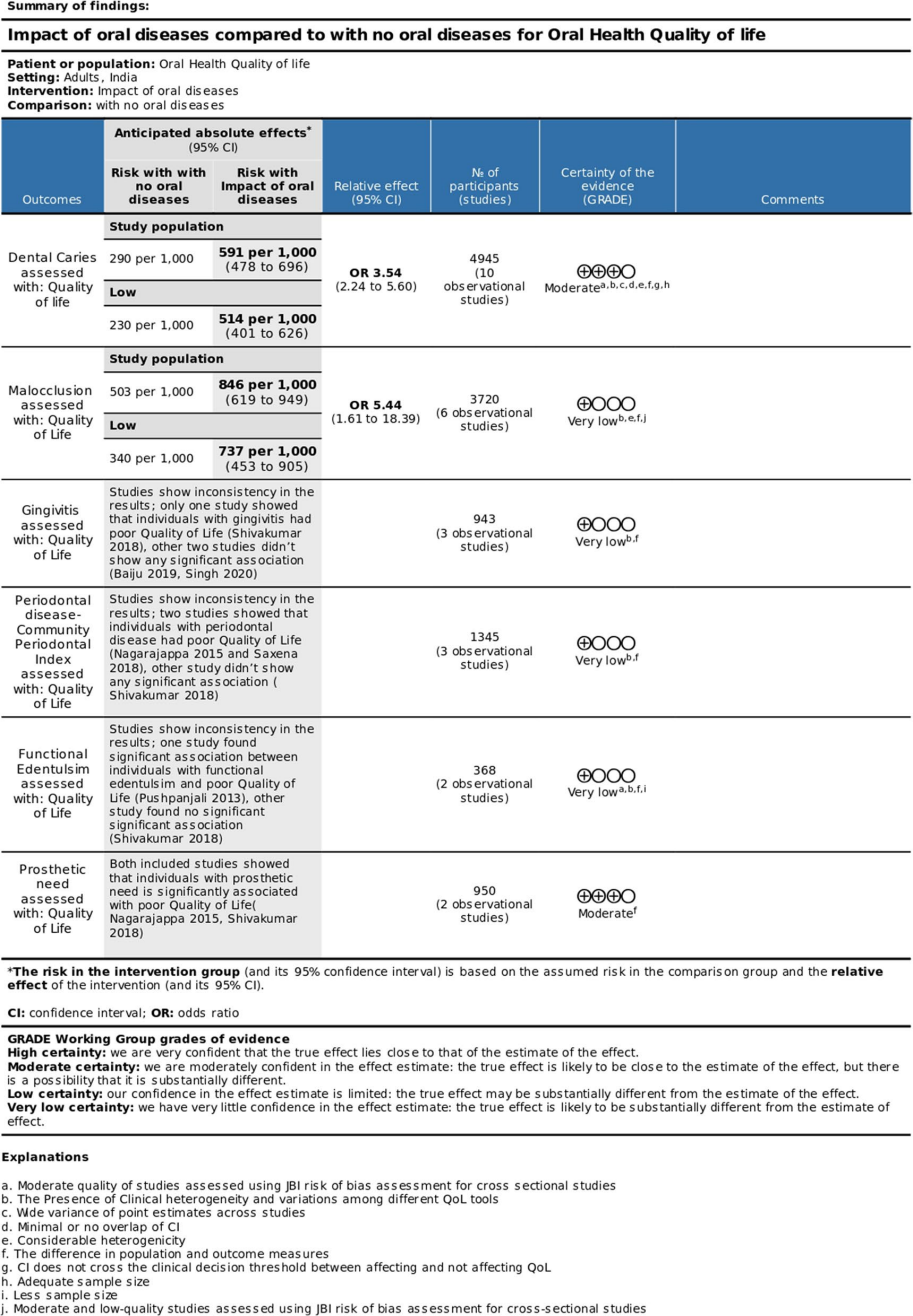
Summary of findings

The two reviewers independently assessed the evidence's certainty using the Grading of Recommendations Assessment, Development and Evaluations (GRADE Approach). The certainty of the evidence for the comparison (oral conditions and Quality of Life) was classified as ‘moderate’ for dental caries and prosthetic need and 'very low' for malocclusion, gingivitis, periodontal disease and functional edentulism. There was a downgrade in the level of evidence due to the methodological quality of the studies, small sample size, and heterogeneity. A summary of the findings table using 'Gradepro' software was generated. Figure 1 shows the summary of the GRADE assessment for the binary outcome.

## Results

### Study inclusion

**Fig. 2:**
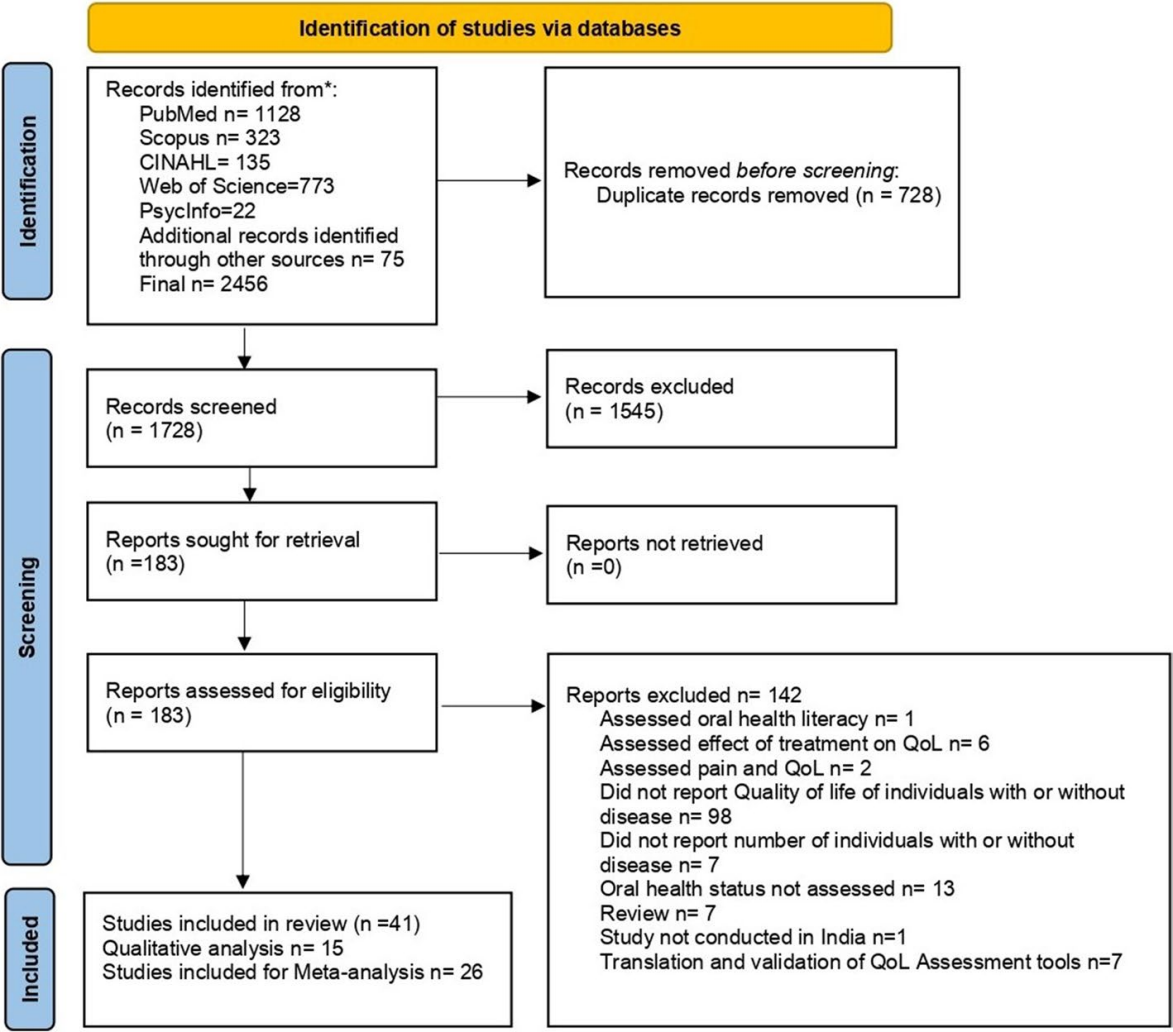
Search results and study selection and inclusion process

A comprehensive and detailed search of the literature yielded 2381 identified records, with 75 additional records found through other resources, for a total sample size of 2456 studies. 728 duplicates were removed from the 2456 articles, leaving 1728 records to be reviewed by title and abstract. After reviewing the titles and abstracts, we determined that 1545 did not meet our eligibility requirements. As a result, 183 articles were retrieved for full-text evaluation, 142 of which were rejected because they did not meet the eligibility criteria. Thus, 41 studies were considered for the systematic review: fifteen studies for qualitative synthesis and twenty-six studies for meta-analysis. The PRISMA flowchart search and review process for study selection and inclusion is depicted in Fig. 2.

### Methodological quality

All the included studies underwent critical appraisal. No studies were excluded based solely on the assessment of methodological quality. Out of 41 studies, 16 studies were of high quality [41–56], 17 studies of medium quality [38, 57–72], and eight studies of low quality [73–80]. Only nine of 41 included studies had a low risk of bias. Most included studies used validated and reliable tools to measure exposure and outcome (Q3 and Q7). While most of the included studies failed to address the confounding issues (Q6). The methodological quality of all 41 publications evaluated is summarised in Table 1.

**Table 1:** Critical appraisal of the included studies

Author, Year	Q1	Q2	Q3	Q4	Q5	Q6	Q7	Q8	Quality Assessment
Abhishek 2014 [73]	N	U	Y	Y	N	N	Y	N	Low quality
Abhishek 2016 [74]	N	U	Y	Y	N	N	Y	N	Low quality
Ajai 2020 [57]	Y	Y	Y	Y	N	N	Y	Y	Medium quality
Appukuttan 2016 [58]	Y	Y	N	N	N	N	Y	Y	Medium quality
Babu 2017 [59]	Y	Y	Y	Y	N	N	Y	Y	Medium quality
Baiju 2019 [38]	Y	Y	Y	U	N	N	Y	Y	Medium quality
Basavaraj 2014 [60]	Y	Y	Y	Y	N	N	Y	Y	Medium quality
Ghanghas 2019 [61]	Y	Y	Y	Y	N	N	Y	Y	Medium quality
Jaggi 2019 [62]	Y	Y	Y	Y	N	N	Y	Y	Medium quality
Jain 2012 [75]	N	N	Y	U	U	U	U	U	Low quality
Joseph 2016 [63]	Y	Y	Y	Y	U	U	Y	Y	Medium quality
Kumar 2015 [41]	Y	Y	Y	Y	Y	Y	Y	Y	High quality
Kumar 2018 [55]	Y	Y	Y	Y	Y	N	Y	Y	High quality
Manjith 2012 [64]	Y	Y	Y	Y	N	N	Y	U	Medium quality
Marina 2019 [76]	U	N	N	N	N	N	N	N	Low quality
Mary 2019 [56]	Y	Y	Y	Y	Y	N	Y	Y	High quality
Marya 2020 [42]	Y	Y	U	Y	Y	Y	Y	Y	High quality
Nagarajappa 2015 [43]	Y	Y	Y	Y	Y	Y	Y	Y	High quality
Neelamana 2020 [65]	U	Y	Y	Y	Y	N	Y	N	Medium quality
Pillai 2015 [44]	Y	Y	Y	Y	Y	Y	Y	Y	High quality
Pushpanjali 2013 [45]	Y	Y	Y	Y	Y	Y	Y	Y	High quality
Rajagopalachari 2015 [46]	Y	U	Y	Y	Y	Y	Y	Y	High quality
Rekhi 2015 [77]	U	U	U	Y	U	U	Y	Y	Low quality
Rekhi 2016 [47]	Y	Y	Y	Y	Y	U	Y	Y	High quality
Rekhi 2018 [66]	U	Y	Y	Y	Y	U	U	Y	Medium quality
Sanadhya 2015 [67]	U	Y	Y	Y	Y	Y	Y	U	Medium quality
Saxena 2018 [48]	Y	Y	Y	Y	Y	Y	Y	Y	High quality
Sharna 2019 [68]	U	Y	Y	Y	U	U	Y	U	Medium quality
Shetty 2013 [78]	U	Y	Y	Y	N	N	U	U	Low quality
Shivakumar 2018 [69]	U	Y	Y	U	Y	U	Y	U	Medium quality
Shyam 2020 [70]	Y	Y	Y	Y	Y	Y	U	U	Medium quality
Siluvai 2015 [49]	Y	Y	Y	N	Y	Y	Y	Y	High quality
Singh 2019 [80]	U	U	U	N	Y	N	N	N	Low quality
Singh 2020 [79]	U	Y	Y	U	N	N	Y	U	Low quality
Singh N 2020 [50]	Y	Y	U	Y	Y	Y	Y	Y	High quality
Sreela 2020 [51]	Y	Y	Y	Y	Y	Y	Y	Y	High quality
Suguna 2020 [71]	U	U	Y	Y	N	N	Y	Y	Medium quality
Thetakala 2018 [52]	Y	Y	Y	Y	Y	Y	Y	Y	High quality
Usha 2012 [53]	Y	Y	Y	Y	Y	Y	Y	Y	High quality
Vinayagamoorthy 2020 [54]	Y	Y	Y	Y	Y	Y	Y	Y	High quality
Yadav 2019 [72]	Y	Y	Y	Y	N	N	Y	Y	Medium quality

Critical appraisal questions:

Q1. Were the criteria for inclusion in the sample clearly defined?

Q2. Were the study subjects and the setting described in detail?

Q3. Was the exposure measured in a valid and reliable way?

Q4. Were objective, standard criteria used for measurement of the condition?

Q5. Were confounding factors identified?

Q6. Were strategies to deal with confounding factors stated?

Q7. Were the outcomes measured in a valid and reliable way?

Q8. Was appropriate statistical analysis used?

Y, Yes: U, Unclear; N, No

### Characteristics of the included studies

Table 2 summarises the details of the current systematic review, which included 41 articles for descriptive analysis. Except for one study by Singh N et al. [49], which was a randomised controlled trial in which the baseline data was considered for the purpose of the analysis, all of the included studies were cross-sectional. The studies included were published between 2012 and 2022, and they were all in English.

**Table 2:** Characteristics of the included studies

Author, Year	State/ India Location of the study	Total sample size (N)	Study population	Age group	Measurement of Quality of Life (QoL)	Measurement of Oral disease
**Dental caries**						
Abhishek 2014 [73]	Karnataka	172	Police Personnel	20–60 years	OHIP-14	WHO-1997
Ajai 2020 [57]	Uttar Pradesh	100	Elderly	> = 60 years	OHIP-14	WHO-1997
Appukuttan 2016 [58]	Tamil Nadu	199	General Population	20–70 years	GOHAI	Not mentioned
Babu 2017 [59]	Karnataka	300	Childrens	2–6 years	ECOHIS	WHO-1997
Baiju RMP 2019 [38]	Kerala	400	Adolescents	15–18 years	OHIA	DMFT
Basavaraj 2014 [60]	Uttar Pradesh	900	Childrens	12–15 years	Child- OIDP	DMFT
Ghanghas 2019 [61]	Haryana	469	Childrens	3–5 years	ECOHIS	deft
Jaggi 2019 [62]	New Delhi	750	Childrens	4–6 years	ECOHIS	WHO- 2013
Jain 2012	Gujarat and Rajasthan	1441	General population	25–54 years	OHIP-14	WHO-1997
Kumar 2015 [41]	Madhya Pradesh	690	Childrens	12–15 years	OIDP	DMFT
Kumar 2018 [55]	Kerala	281	Childrens	12 years	COHIP	DMFT
Nagarajappa 2015 [43]	Rajasthan	800	General Population	17–24 years	OIDP	WHO-1997
Rekhi 2015 [77]	Uttarakhand	368	Elderly	> = 60 years	GOHAI	WHO-1997
Rekhi 2018 [66]	New Delhi	500	Elderly	> = 60 years	GOHAI	DMFT
Saxena 2018 [48]	Uttar Pradesh	414	School teachers	> 20 years	OIDP	WHO-1997
Sharna 2019 [68]	Tamil Nadu	238	Childrens	6–72 mths	ECOHIS	PUFA
Shivakumar 2018 [69]	Maharashtra	150	Elderly	> = 60 years	GOHAI	WHO-1997
Singh N 2020 [50]	Uttar Pradesh	200	Childrens	3–5 years	Michigan oral health related quality of life scale	deft-Index
Sreela 2020 [51]	Kerala	1552	General Population	18–74 years	OHIP-14	WHO-2013
Usha 2012 [53]	Karnataka	900	Childrens	12–15 years	OIDP	DMFT
**Gingivitis**						
Appukuttan 2016 [58]	Tamil Nadu	199	General Population	20–70 years	GOHAI	Not mentioned
Baiju RMP 2019 [38]	Kerala	400	Adolescents	15–18 years	OHIA	Gingival index
Marya 2020 [42]	Haryana	1200	Elderly	> = 60 years	GOHAI	WHO-2013
Shivakumar 2018 [69]	Maharashtra	150	Elderly	> = 60 years	GOHAI	WHO-1997
Singh 2020 [79]	Uttar Pradesh	395	Childrens	11–14 years	CPQ 11–14	Gingival index
Sreela 2020 [51]	Kerala	1552	General Population	18–74 years	OHIP-14	WHO-2013
**Periodontal Disease- Loss of Attachment**						
Abhishek 2016 [74]	Karnataka	172	Police Personnel	20–60 years	OHIP-14	WHO-1997
Marya 2020 [42]	Haryana	1200	Elderly	> = 60 years	GOHAI	WHO-2013
Nagarajappa 2015 [43]	Rajasthan	800	General Population	17–24 years	OIDP	WHO-1997
Rajagopalachari 2015 [46]	Kerala	212	General Population	24–60 years	OHIP-14	WHO-1997
Rekhi 2016 [47]	New Delhi	500	Elderly	> = 60 years	GOHAI	WHO-1997
Sanadhya 2015 [67]	Rajasthan	1200	General population	20–79 years	OHIP-14	WHO-1997
Saxena 2018 [48]	Uttar Pradesh	414	School teachers	> 20 years	OIDP	WHO-1997
Sreela 2020 [51]	Kerala	1552	General Population	18–74 years	OHIP-14	WHO-2013
Yadav 2019 [72]	Haryana	450	General Population	30–60 years	OHIP-14	Clinical Attachment Loss
**Periodontal Disease -Community Periodontal Index**						
Abhishek 2016 [74]	Karnataka	172	Police Personnel	20–60 years	OHIP-14	WHO-1997
Ajai 2020 [57]	Uttar Pradesh	100	Elderly	> = 60 years	OHIP-14	WHO-1997
Marya 2020 [42]	Haryana	1200	Elderly	> = 60 years	GOHAI	WHO-2013
Nagarajappa 2015 [43]	Rajasthan	800	General Population	17–24 years	OIDP	WHO-1997
Rajagopalachari 2015 [46]	Kerala	212	General Population	24–60 years	OHIP-14	WHO-1997
Rekhi 2016 [47]	New Delhi	500	Elderly	> = 60 years	GOHAI	WHO-1997
Sanadhya 2015 [67]	Rajasthan	1200	General population	20–79 years	OHIP-14	WHO-1997
Saxena 2018 [48]	Uttar Pradesh	414	School teachers	> 20 years	OIDP	WHO-1997
Shivakumar 2018 [69]	Maharashtra	150	Elderly	> = 60 years	GOHAI	WHO-1997
Sreela 2020 [51]	Kerala	1552	General Population	18–74 years	OHIP-14	WHO-2013
**Malocclusion**						
Appukuttan 2016 [58]	Tamil Nadu	199	General Population	20–70 years	GOHAI	Not mentioned
Babu 2017 [59]	Karnataka	300	Childrens	2–6 years	ECOHIS	WHO-1997
Baiju RMP 2019 [38]	Kerala	400	Adolescents	15–18 years	OHIA	DAI
Basavaraj 2014 [60]	Uttar Pradesh	900	Childrens	12–15 years	Child- OIDP	DAI
Manjith 2012 [64]	Puducherry	200	Childrens	11–15 years	OHIP- 14	IOTN
Mary 2019 [56]	Tamil Nadu	710	Adolescents	17–23 years	OHIP- 14	IOTN
Nagarajappa 2015 [43]	Rajasthan	800	General Population	17–24 years	OIDP	WHO-1997
Siluvai 2015 [49]	Karnataka	900	General Population	13–19 years	OHIP-14	DAI
Singh 2019 [80]	New Delhi	520	Childrens	12–15 years	OHIP-14	IOTN
Vinayakamoorthy 2020 [54]	Karnataka	768	Childrens	12–15 years	FIS	DAI
**Dental Fluorosis**						
Nagarajappa 2015 [43]	Rajasthan	800	General Population	17–24 years	OIDP	WHO-1997
Shyam 2020 [70]	Haryana	2200	Childrens	11–14 years	CPQ 11–14	TFI
**Bruxism**						
Suguna 2020 [71]	Tamil Nadu	72	Childrens	6–12 years	OHIP-14	Interview
Thetakala 2018 [52]	Karnataka	212	General Population	> = 18 years	OHIP-14	criteria of American Academy of Sleep Medicine
**Functional Edentulism**						
Neelamana 2020 [65]	Kerala	280	Elderly	> 60 years	GOHAI	WHO-2013
Pushpanjali 2013 [45]	Karnataka	218	Elderly	> 60 years	OHIP-14	WHO-1997
Rekhi 2015 [77]	Uttarakhand	368	Elderly	> = 60 years	GOHAI	WHO-1997
Rekhi 2018 [66]	New Delhi	500	Elderly	> 60 years	GOHAI	WHO-1997
Shetty 2013 [78]	Maharashtra	110	Elderly	> = 60 years	GOHAI	WHO-1997
Shivakumar 2018 [69]	Maharashtra	150	Elderly	> = 60 years	GOHAI	WHO-1997
**Edentulism**						
Ajai 2020 [57]	Uttar Pradesh	100	Elderly	> = 60 years	OHIP-14	WHO-1997
Appukuttan 2016 [58]	Tamil Nadu	199	General Population	20–70 years	GOHAI	Not mentioned
Marina 2019 [76]	Tamil Nadu	300	Elderly	60–85 years	WHOQOL-old	Not mentioned
Shivakumar 2018 [69]	Maharashtra	150	Elderly	> = 60 years	GOHAI	WHO-1997
**Prosthetic need**						
Joseph 2016 [63]	Kerala	539	Elderly	> = 60 years	OHIP-14	WHO-1997
Nagarajappa 2015 [43]	Rajasthan	800	General Population	17–24 years	OIDP	WHO-1997
Pillai 2015 [44]	New Delhi	946	Elderly	> 60 years	GOHAI	WHO-1997
Rekhi 2018 [66]	New Delhi	500	Elderly	> 60 years	GOHAI	WHO-1997
Shetty 2013 [78]	Maharashtra	110	Elderly	> = 60 years	GOHAI	WHO-1997
Shivakumar 2018 [69]	Maharashtra	150	Elderly	> = 60 years	GOHAI	WHO-1997

Functional Edentulism- < 20 remaining teeth

Edentulism – Complete or partial missing teeth

^*^COHIP Child Oral Health Impact Profile, *CPQ* Child Perceptions Questionnaire, *deft* decayed, extracted, filled teeth, *DAI* Dental Aesthetic Index, *DMFT* Decayed Missing Filled Teeth, *ECOHIS* Early Childhood Oral Health Impact Scale, *FIS* Family Impact Scale, *GOHAI* Geriatric Oral Health Assessment Index, *IOTN* Index of Orthodontic Treatment Need, *OHIA* Oral Health Impacts in Adolescents, *OHIP* Oral Health Impact Profile, *OIDP* Oral Impact on Daily Performance, *PUFA* Pulpal involvement, Ulceration, Fistula, Abscess, *TFI Thylstrup*–*Fejerskov* I*ndex, WHO* World Health Organization, *WHOQoL* World Health Organization Quality of Life

According to age group, 15 studies assessed OHRQoL among childrens [41, 50, 53–55, 59–62, 64, 68, 70, 71, 79, 80], two was in adolescents [38, 56], 12 were in elderly population [42, 44, 45, 47, 57, 63, 65–67, 76–78] and 12 were in general population [43, 46, 48, 49, 51, 52, 58, 67, 72–75].

Studies also evaluated the impact of oral conditions over OHRQoL, classified according to exposure to one or more oral conditions, dental caries, gingivitis, periodontal disease, edentulism, malocclusion, dental fluorosis, bruxism and prosthetic need. Studies related to dental caries are (*n* = 20) [38, 41, 43, 48, 50, 51, 53, 55, 57–62, 66, 68, 69, 73, 75, 77], Gingivitis (*n* = 6) [38, 42, 51, 58, 69, 79], Periodontal Disease assessed by Loss of Attachment (*n* = 9) [42, 43, 47, 48, 51, 67, 69, 72, 74], Periodontal Disease assessed by Community Periodontal Index (*n* = 10) [42, 43, 46–48, 51, 57, 67, 69, 74], malocclusion (*n* = 10) [38, 43, 49, 54, 56, 58–60, 64, 80], Dental fluorosis (*n* = 2) [43, 70], Bruxism (*n* = 2) [52, 71], Functional Edentulism/Edentulism (*n* = 9) [45, 57, 58, 65, 66, 69, 76–78], Prosthetic need (*n* = 6) [43, 44, 63, 66, 69, 78].

The quality-of-life measurement instruments used in the different studies was as follows; CPQ11-14 [70, 79], ECOHIS [59, 61, 62, 68], OHIP-14 [45, 46, 49, 51, 52, 56, 57, 63, 64, 67, 71–75, 80], OIDP [41, 43, 48, 53, 60], OHIA [38], WHOQoL [76], Michigan Oral Health related quality of life scale [50], FIS [54], Child Oral Health Impact Profile (COHIP) [55] and GOHAI [42, 44, 47, 58, 65, 66, 69, 77, 78], the most frequent being OHIP-14 (*n* = 16) and GOHAI (*n* = 9).

### Dental caries and OHRQoL

Twenty studies with a total sample of 10,650 individuals assessed the relationship between dental caries and OHRQoL. Eleven studies assessed dental caries with binary outcome and nine studies with continuous measures. Four studies evaluated OHRQoL with GOHAI or OHIP-14, and dental caries was assessed with WHO criteria.

a Dental caries and QoL (Binary outcome)

**Fig. 3:**
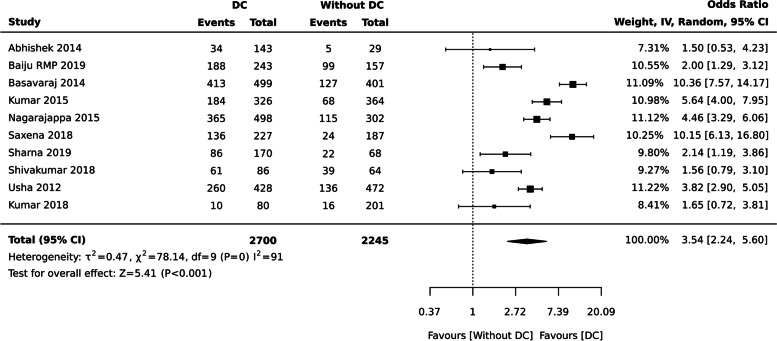
Dental caries and QoL

Individuals with dental caries have nearly four times the chances of having a poor OHRQoL compared to those without dental caries [ OR:3.54 (95% CI 2.24, 5.60), ten studies, 4945 participants], but there was substantial heterogeneity (91%) across the studies (Fig. 3).

b Dental caries and QoL (Continuous data measured by OHIP-14/ ECOHIS/ Michigan Oral Health Quality of Life)

**Fig. 4:**
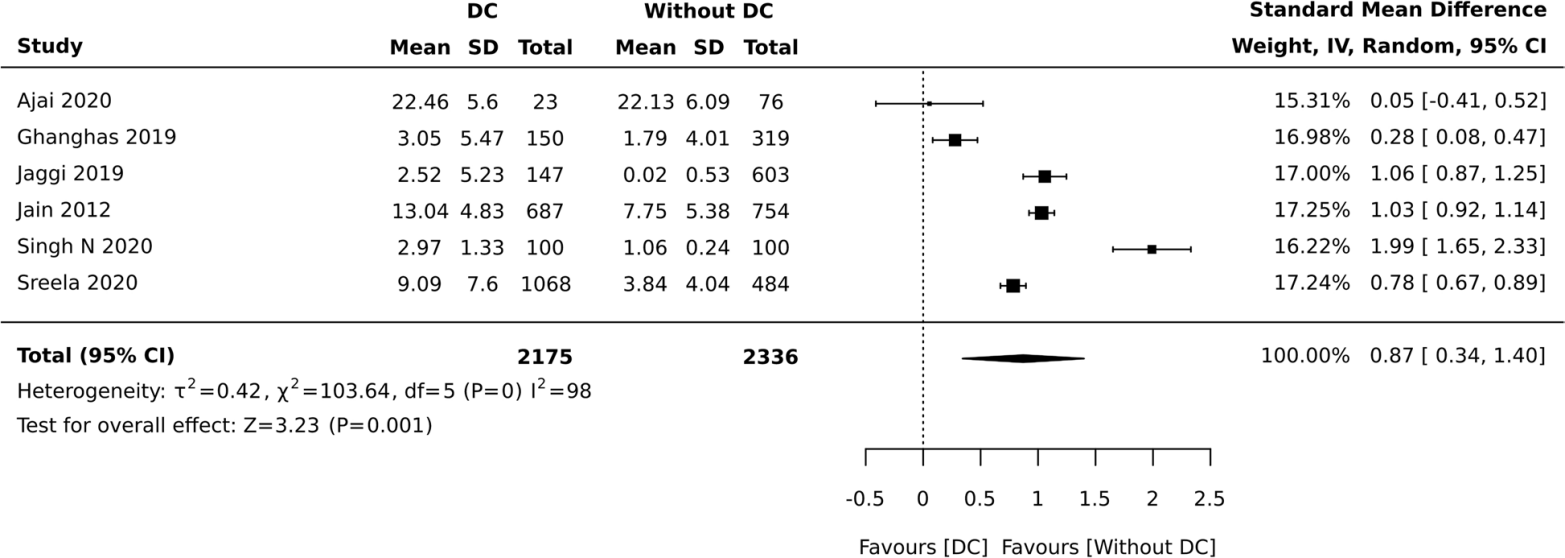
Dental caries and QoL measured by OHIP-14, ECOHIS, and Michigan Oral Health Quality of Life

OHRQoL favoured individuals without dental caries when assessed using OHIP-14, ECOHIS, and Michigan Oral Health Quality of Life [SMD: 0.87 (95% CI 0.34, 1.40), six studies, 4511 participants], I^2^ = 98% (Fig. 4). Babu et al. [59] with 300 individuals was not considered for meta-analysis as one of event in the binary outcome was zero.

iii Dental caries and QoL (GOHAI)

All the three studies [58, 66, 77] were QoL assessed by the GOHAI showed that individuals with decayed teeth is associated with the poor QoL (*P* < 0.05).

### Gingivitis and OHRQoL

Seven studies with 3679 individuals assessed the impact of gingivitis and OHRQoL.

a Gingivitis and QoL (Binary outcome)
  Of the 3 studies, only one study reported a significant association of gingivitis on OHRQoL [OR 1.39 (1.09, 1.67)] [69] and other two studies found no significant association [38, 79].
b Gingivitis and QoL (Continuous data measured by GOHAI)
  Appukuttan et, al. 2016 [58] found that individuals with gingivitis had poor OHRQoL (*P*<0.05) and other study reported no significant association (*P*=0.08) [42].
c Gingivitis and QoL (Continuous data measured by OHIP-14)
  Individuals with gingivitis had no impact on OHRQoL when assessed with OHIP-14 (*P*= 0.0762) [51].

### Periodontal Disease assessed by Loss of Attachment (LOA) and OHRQoL

Nine studies with 6289 individuals assessed the relationship between periodontal disease assessed by LOA and OHRQoL.

a Periodontal disease- LOA and QoL (Continuous data measured by OHIP-14/ OIDP)

**Fig. 5:**
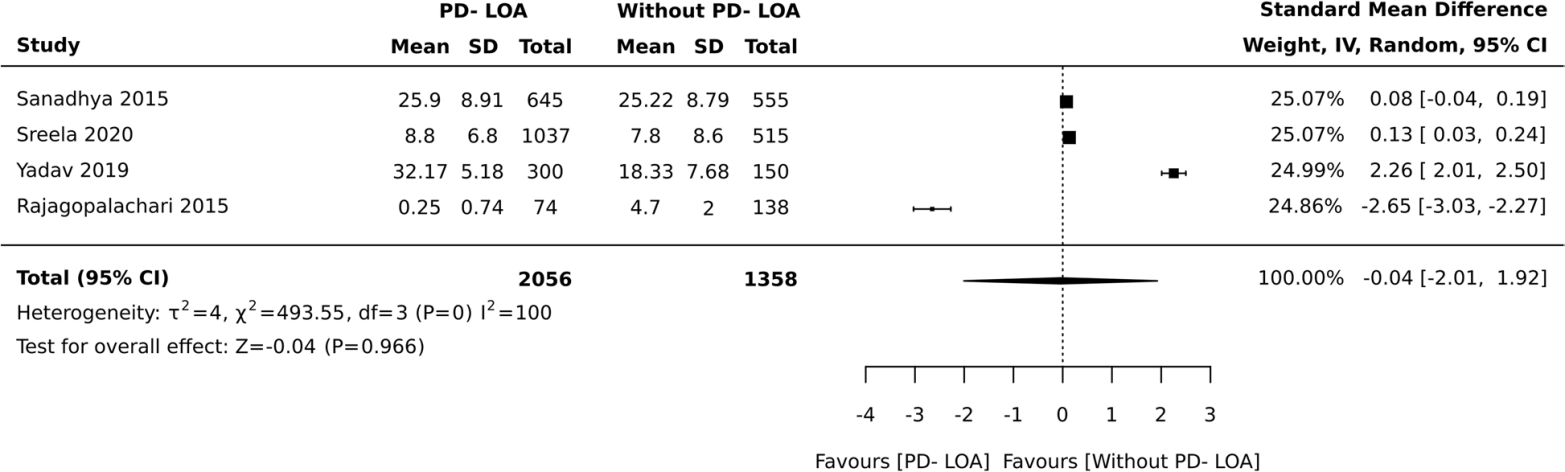
Periodontal disease- Loss pf Attachment measured by OHIP-14 and OIDP

No difference in OHRQoL between the groups was observed for studies with continuous measures, when OHRQoL assessed with OHIP-14 and OIDP [ SMD: -0.04 (95% CI -2.01, 1.92), four studies, 3414 participants], I^2^ = 100% (Fig. 5).

b Periodontal disease- LOA and QoL (Binary outcome)

Two studies found there is a significant association between individuals with periodontal disease and OHRQoL [48, 74] and another study by Nagarajappa et al. 2015 denied the association (*P*=0.687) [43]

iii Periodontal disease- LOA and QoL (Continuous data measured by GOHAI)

One study reported that periodontal diseases had a significant negative impact on OHRQoL [47] and other study by Marya et al. 2020 failed to find a significant association [42].

### Periodontal Disease assessed by Community Periodontal Index (CPI) and OHRQoL

Overall, ten studies with 6,300 individuals evaluated the impact between periodontal disease assessed by CPI and OHRQoL. In most studies, OHIP-14 is used to assess the OHRQoL and WHO criteria to measure Periodontal Disease.

a Periodontal disease- CPI and QoL (Continuous data measured by OHIP-14/ OIDP)

**Fig. 6:**
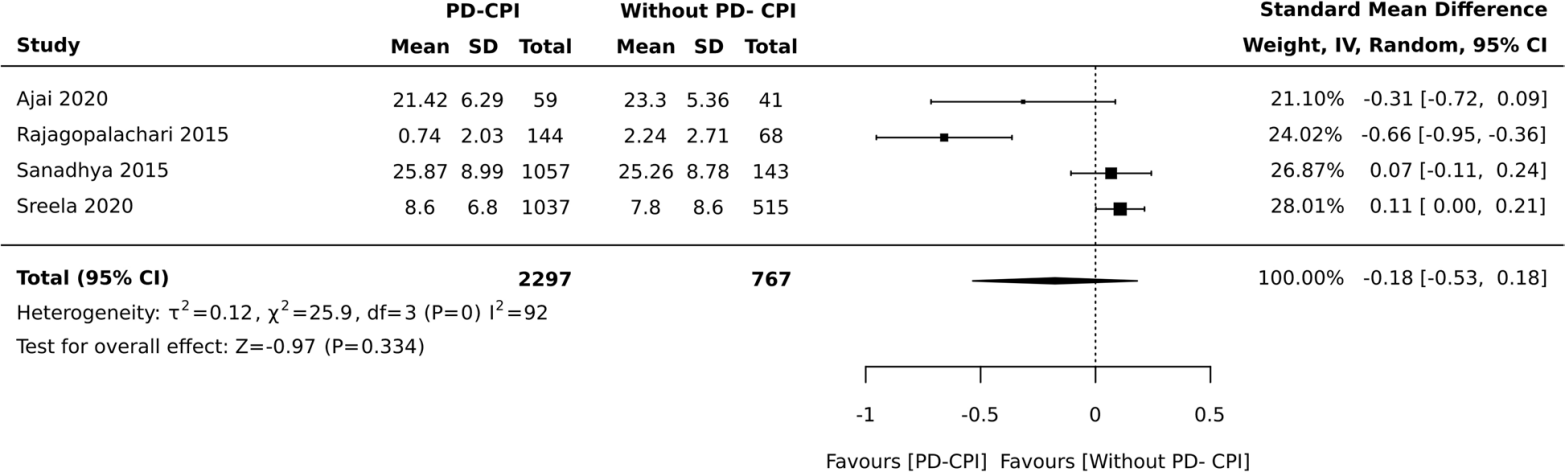
Periodontal disease- Community Periodontal Disease measured by OHIP-14 and OIDP

There was no difference between two groups when OHRQoL assessed using OHIP- 14 and OIDP scale [ SMD: -0.18 (95% CI -0.53, 0.18), four studies, 3064 participants], I^2^= 92% (Fig. 6).

b Periodontal disease- CPI and QoL (Binary outcome)

Of the four studies, 3 studies found that individuals with periodontal disease had a negative impact on the OHRQoL [43, 48, 74] and a study by Shivakumar et al. 2018 failed to establish the association [69].

iii Periodontal disease- CPI and QoL (Continuous data measured by GOHAI)

Community Periodontal Index scores showed a negative correlation with OHRQoL [47]. No significant relation was seen between OHRQoL periodontal pocket (*P* > 0.05) [42].

### Functional Edentulism and OHRQoL

Six studies with 1504 individuals assessed the relationship between functional edentulism and OHRQoL.

a Functional edentulism and QoL (Continuous data measured by GOHAI)

**Fig. 7:**
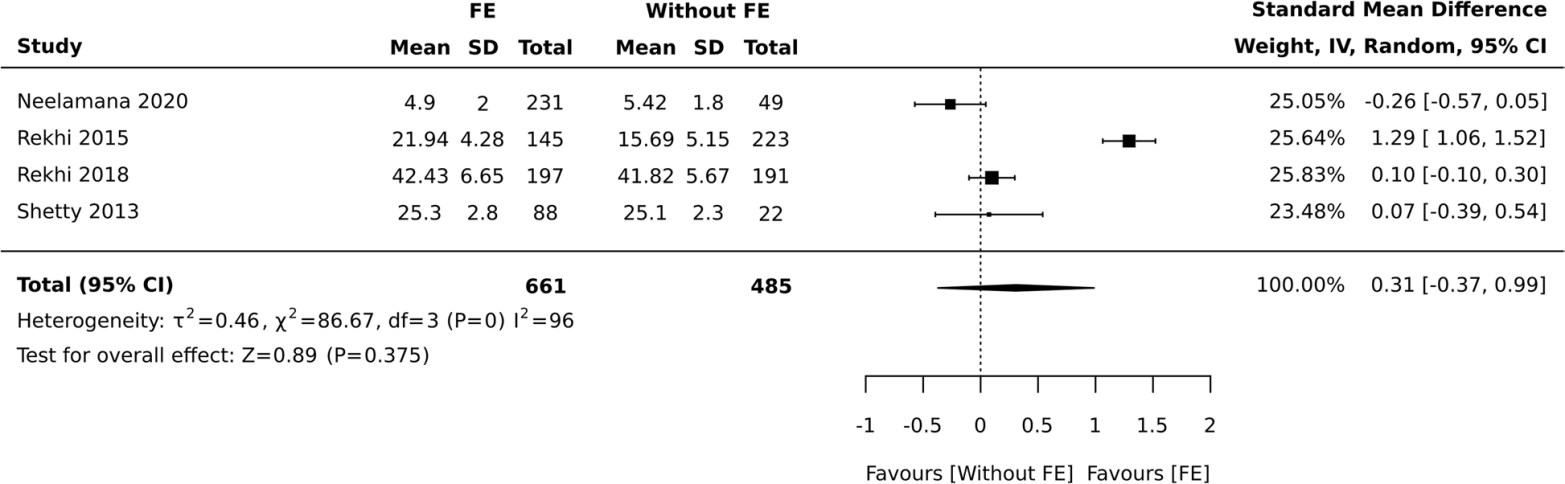
Functional edentulism and QoL measured by GOHAI

No significant association on OHRQoL was found between individual’s with and without functional edentulism when QoL measured by GOHAI [ SMD: 0.31 (95% CI -0.37, 0.99), four studies, 1146 participants], I^2^= 96% (Fig. 7).

b Functional edentulism and QoL (Binary outcome)

One study found a significant association between functional edentulism [45] and OHRQoL; another study failed to prove the association [69].

### Edentulism and OHRQoL

Four studies with 749 individuals evaluated the relationship between edentulism and OHRQoL.

a Edentulism and QoL (Binary outcome)

Edentulism does not have any impact on the OHRQoL [69].

b Edentulism and QoL (Continuous data measured by GOHAI/ WHOQoL)

One study reported that edentulism negatively influenced quality of life [76]. The study by Appukuttan et al. 2016 reported that edentulism did not affect quality of life [58].

iii Edentulism and QoL (Continuous data measured by OHIP-14)

A study by Ajai et al. 2020. There is no significant association on OHRQoL between individuals with and without edentulism on OHRQoL [57].

### Malocclusion and OHRQoL

Ten studies with 5687 individuals assesses the impact of malocclusion on OHRQoL.

a Malocclusion and QoL (Binary outcome)

**Fig. 8:**
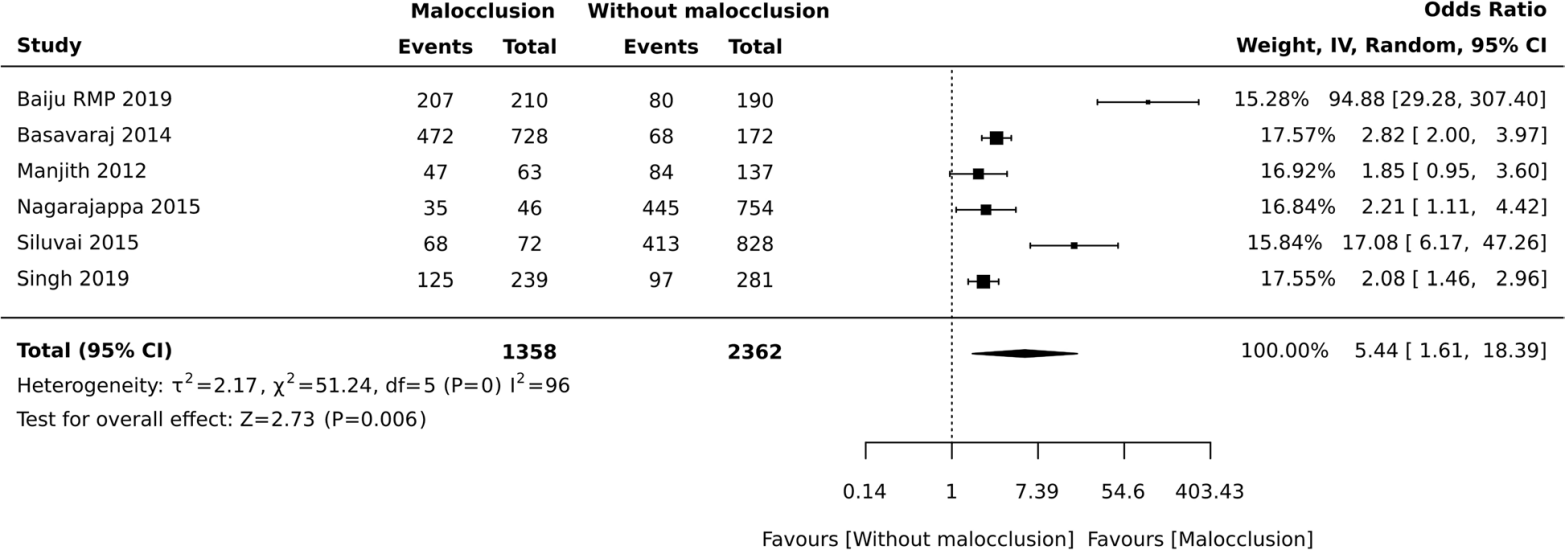
Malocclusion and QoL

The statistically significant difference was observed between two groups: individuals with malocclusion and without malocclusion [ OR: 5.44 (95% CI 1.61, 18.39), six studies, 3720 participants], I^2^= 96% (Fig. 8). Babu et al. [59] with 300 individuals was also not considered for meta-analysis as one event in the binary outcome was zero.

b Malocclusion and QoL (Continuous data measured by GOHAI)

There is no significant difference on OHRQoL between individuals with and without malocclusion [58].

iii Malocclusion and QoL (continuous data measured by OHIP-14 / ECOHIS/ FIS)

Malocclusion had a significant negative influence on the OHRQoL [54, 56].

### Dental Fluorosis and OHRQoL

Two studies with 3000 individuals assesses the impact of dental fluorosis on OHRQoL.

a Dental fluorosis and QoL (continuous data measured by OIDP/ CPQ 11-14)

There is a significant difference between individuals with and without fluorosis on the OHRQoL [43, 70].

### Bruxism and OHRQoL

Two studies with 284 individuals assesses the impact of bruxism on OHRQoL.

a Bruxism and QoL (continuous data measured by OHIP-14)

Bruxers have poor OHRQoL than non-bruxers [52, 71].

### Prosthetic need and OHRQoL

Six studies with 3045 individuals assesses the impact of prosthetic need on OHRQoL.

a Prosthetic need and QoL (Binary outcome)
  Individuals with prosthetic need is significantly associated with the poor OHRQoL [43, 69].
b Prosthetic need and QoL (continuous data measured by GOHAI)
  Of the three studies, only one study found that there is no significant difference between indivduals with prosthetic need and without prosthetic need [78] and other two studies proved the association [44, 66].
c Prosthetic need and QoL (continuous data measured by OHIP-14)
  The prosthetic need was significantly related to various components of OHRQOL [63].

### Subgroup analysis

1. Dental caries
  a Based on the scale (direction of the scores) used to measure OHRQoL (Binary outcome)

**Fig. 9:**
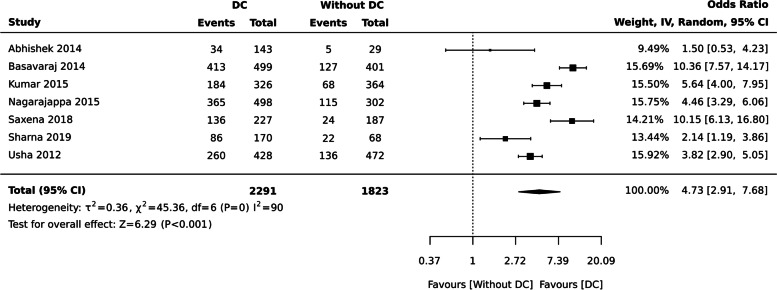
Sub group analysis based on scales for dental caries

People with dental caries had poor OHRQoL [ OR: 4.73 (95% CI 2.91, 7.68), seven studies, 4114 participants], I^2^= 90% (Fig. 9).

b Based on the age group for adolescence (10–19 years) (Binary outcome)

**Fig. 10:**
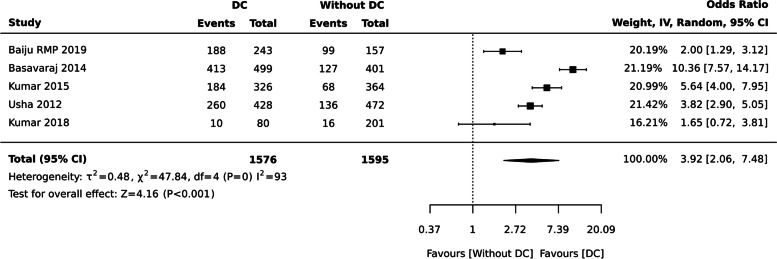
Subgroup analyis based on age group for dental caries

Individuals with dental caries had poor OHRQoL [ OR: 3.92 (95% CI 2.06, 7.48), five studies, 3171 participants], I^2^= 93% (Fig. 10).

iii Based on the age group for children (3–5 years) (Continuous data- measured by ECOHIS, and Michigan Oral Health Quality of Life)

Early childhood caries is significantly associated with the poor OHRQoL [50, 61, 62].

2. Periodontal disease- CPI and QoL (Continuous data measured by OHIP-14 and OIDP)

No significant association between periodontal disease measured by Community Periodontal Index and OHRQoL for the age group between 20 to 79 years [46, 51, 67].

Table 3 Summarizes the description of outcome measurement and oral health conditions with measure of effect.

**Table 3:** Description of outcome measurement and oral health conditions with measure of effect

Exposure	Based on OHRQoL instruments	N	No. of Individuals with Oral Diseases	No. of Individuals without oral diseases	Number of studies	Pooled Estimate	95% CI		Grading of evidence
							**LL**	**UL**	
**a. Dichotomous data**									
Dental caries	Overall	4945	2700	2245	10	OR 3.54	2.24	5.60	Medium quality
Malocclusion	Overall	3720	1358	2362	6	OR 5.44	1.61	18.39	Medium quality
**b. Continuous data**									
Dental caries	OHIP-14, ECOHIS and Michigan oral health-related QoL	4511	2175	2336	6	SMD 0.87	0.34	1.40	Medium quality
Periodontal Disease – Loss of attachment	OHIP-14 and OIDP	3414	2056	1358	4	SMD -0.04	-2.01	1.92	High quality
Periodontal Disease—Community Periodontal Index	OHIP-14 and OIDP	3064	2297	767	4	SMD -0.18	-0.53	0.18	High quality
Functional edentulism	GOHAI	1146	661	485	4	SMD 0.31	-0.37	0.99	Medium quality

## Discussion

The purpose of this study was to conduct a systematic analysis of the effect of oral conditions on OHRQoL in Indians. To the best of our knowledge, no systematic review with meta-analysis has assessed the impact of oral conditions on quality of life; this is the first. Because of the implications for oral health disparities and access to care, this evidence is required for healthcare decision-making. Given the disparities in the availability, quality, and access to oral health care in India, comparing OHRQoL across groups may help patients, healthcare providers, and policymakers make better decisions [35].

The review demonstrated that the experience of poor OHRQoL is higher among individuals with dental caries, and malocclusion. Pooled evidence confirms that these oral conditions hurt the general state of well-being.

Meta-analysis was performed separately for binary outcome and continuous measures, considering the odds ratio and standard mean difference. OHRQoL assessment scales has different interpretation based on the scores. For example, for GOHAI, COHIP, and WHOQoL, as the score increases, there is an increase on on OHRQoL. Nevertheless, OHIP-14, OIDP, CPQ 11-14, ECOHIS, FIS and Michigan oral health quality of life decreases OHRQoL as the score increases. Hence, for continuous results, scales were divided into two groups based on the direction of the score and a meta-analysis was performed. For binary outcome, all the scales were considered together for meta-analysis irrespective of the direction of the score.

### Dental caries and OHRQoL

Dental caries was found to be associated with impaired OHRQoL in the current review. This is consistent with findings of the systematic review by Nora et al. [30] and Zaror et al. [81]. Impact of dental caries on the OHRQoL increases, primarily due to pain and damage to aesthetics, which affect individual social interactions. Moreover, severe dental caries can result in missing school days and having more significant financial expenditures, negatively impacting the OHRQoL of the children's families. Studies included in this systematic review used different caries diagnostic indexes ( dmft/ DMFT, PUFA, WHO criteria), which can interfere with the pooled data summary. Furthermore, there are variations in the criteria to define the severity of dental caries (DMFT > 1, only considered decayed component etc.). This lack of tool standardisation to measure both exposure and outcome can affect the findings.

### Gingivitis, periodontal disease, and OHRQoL

We have found that Periodontal diseases had no impact on OHRQoL, which is similar to the findings by Wong et al. [31].

Dose–response effect OHRQoL.

The periodontal disease assessment is based on an ordinal scale, and periodontal disease was considered as present for: score 1(bleeding), score 2; calculus present, score3; shallow pocket, score 4; deep pockets. OHRQoL was affected differently depending on the severity of the disease, with severe periodontitis showing a more pronounced adverse effect than mild to moderate periodontitis. OHRQoL is primarily impacted by the esthetic and functional elements of periodontitis. Compared to periodontitis, gingivitis has a smaller effect on OHRQoL, with its main effects being pain, toothbrushing challenges.

### Malocclusion, dental fluorosis, Bruxism and OHRQoL

Our results concluded that there is a significant difference between the two groups concerning malocclusion, affecting quality of life with regard to the appearance of their dentition, self-esteem related to oral health, and interaction with peers. Findings from our study is consistent with the study by Kragt et, al. 2016 [82]. The association of malocclusion and OHRQOL can be due to long-term untreated malocclusions that can result in temporomandibular disorders or trauma. Malocclusion can also results in functional problems like problems with speaking, mastication and subsequent problems restricted food choice [82].

Dental fluorosis rarely causes oral symptoms unless co-morbid disorders such dental caries, enamel fracture, attrition, and dentin hypersensitivity are present, its impact is mainly perceived on a person's assessment of their appearance. Bruxism is associated with the OHRQoL as it can increase the temporomandibular joint load, resulting in signs and symptoms of temporomandibular disorders.

### Edentulism, prosthetic need, and OHRQoL

OHRQoL is negatively associated with prosthetic need. Because partially edentulous patients with denture deficiencies face increased cognitive challenges, such as eating, speaking, avoiding smiling, and other psychological and societal consequences, these significant differences in the experience of oral impacts were expected.

### Heterogeneity

Heterogeneity was more than 90% since different tools were used to measure both exposure (oral conditions) and outcome (OHRQoL). This review included studies of a wide range of age groups and different populations (within country variation), which might have contributed to clinical heterogeneity. Lack of attention to methodological aspects- such as identification of confounding factors, measurement of exposure, and outcome- may have compromised the studies' validity and methodological heterogeneity.

### Implications for research and practice

The tools assessing the OHRQoL need standardisation, more explicitly validated to the local population for being used. This is because the people's perceptions vary for oral conditions among the individuals, thereby attributing heterogeneity. The categorisation of the disease condition does not reflect the pathological process since oral conditions, especially periodontal disease, are chronic and cross-sectional studies cannot establish the temporal relationship.

### Strengths and limitations

This review included all the oral conditions and comprehensively assessed both binary outcome and continuous measures. Each study had a distinct methodology, depending on the age group, the criteria used to diagnose oral disorders, the instrument used to measure OHRQoL, and the association measures that were reported in the research. Only few studies were identified for oral conditions such as gingivitis, periodontitis, edentulism, fluorosis, bruxism, and prosthetic need. Classifying oral conditions, especially periodontal disease, is ambiguous and unable to relate to the quality-of-life assessment. Meta- analysis was performed based on the direction of scores of Quality-of-Life instruments, without considering the cut off value, as criteria defining quality of life and score ranges of each scales were different. Another limitation was that, Even though a wide range of oral conditions has been included in the study, dental trauma was not included in the study.

## Conclusion

Despite the different definitions of the exposures and variety of instruments used to measure OHRQoL, the review demonstrates that the experience of poor quality of life is substantially higher among individuals with dental caries and malocclusion. Due to the poor methodological quality of the research, the limited sample size, and the variability of the included studies, the evidence was low.

### Supplementary Information

**Additional file 1: S1. **Search strategy.

**Additional file 2: Appendix II.** Studies ineligible following full- text review.

## Data Availability

Data sharing is not applicable to this article as no datasets were generated or analysed during the current study.

## Abbreviations

Child-OIDP: Child Oral Impact on Daily Performances
COHIP: Child Oral Health Impact Profile
CPQ11-14: Child Perceptions Questionnaire for children aged 11 to 14 years
DAI: Dental Aesthetic Index
DIDL: Dental Impact on Daily Living
DIP: Dental Impact profile
DMFT/ dmft: Decayed, Missing, Filled Tooth
ECOHIS: Early Childhood Oral Health Impact Scale
FIS: Family Impact Scale
GOHAI: Geriatric Oral Health Assessment Index
GRADE Approach: Grading of Recommendations Assessment, Development and Evaluations
HRQoL: Health Related Quality of Life
IOTN: Index of Orthodontic Treatment Need
OHIA: Oral Health Impact in Adolescents
OHIP-14: Oral Health Impact Profile for 14 years
OHRQoL: Oral Health-Related Quality of Life
OIDP: Oral Impacts on Daily Performances
PRISMA: Preferred Reporting Items for Systematic Reviews and Meta-Analysis
PROSPERO: Prospective International Register of Systematic Reviews
PUFA: Pulpal involvement, Ulceration, Fistula, Abscess;
QoL: Quality of Life
SIDD: Social Impact on Dental Disease
SOHO: Scale of Oral Health Outcomes
TDI: Traumatic Dental Injuries
TFI: *Thylstrup*–*Fejerskov* I*ndex*
WHO: World Health Organization
WHOQoL: World Health Organization Quality of Life
