# Supplementary material for: Impact of oral conditions on oral health-related quality of life among Indians- a systematic review and Meta-analysis
Source: Health Qual Life Outcomes. 2023 Aug 31;21:102. doi: 10.1186/s12955-023-02170-6 (PMC10470255; doi:10.1186/s12955-023-02170-6)
Supplement: Supplementary file 1 — Additional file 1: S1. Search strategy. [file 12955_2023_2170_MOESM1_ESM.docx]

**S1: Search strategy**

Details of searches were performed

1. PubMed- up to July 23, 2022
2. Scopus- up to July 23, 2022
3. CINAHL- up to July 23, 2022
4. Web of Science- up to July 23, 2022
5. PsycInfo- up to July 23, 2022
6. Google scholar- up to July 23, 2022

| **Sl.No** | **Data base** | **Total reference retrieved retrived** |
| --- | --- | --- |
| 1. | **PubMed**  "india*"[All Fields] AND (("Value of Life"[All Fields] OR "Quality of Life"[All Fields] OR "Quality-Adjusted Life Years"[All Fields] OR "daly*"[All Fields] OR "qaly*"[All Fields] OR "HRQOL"[All Fields] OR "QoL"[All Fields] OR "Quality of Life"[All Fields] OR "Dental Impact"[All Fields] OR "Social Impact"[All Fields] OR "Health Impact"[All Fields] OR "COHQOL"[All Fields] OR "CHILD-OIDP"[All Fields] OR "OQLQ"[All Fields] OR "OHQoL"[All Fields] OR "DIDL"[All Fields] OR "ECOHIS"[All Fields] OR "CPQ"[All Fields] OR "OHIP"[All Fields] OR "GOHAI"[All Fields]) AND ("oral health"[MeSH Terms] OR ("oral"[All Fields] AND "health"[All Fields]) OR "oral health"[All Fields] OR ("mouth diseases"[MeSH Terms] OR ("mouth"[All Fields] AND "diseases"[All Fields]) OR "mouth diseases"[All Fields]) OR "tooth*"[All Fields] OR "teeth*"[All Fields] OR ("dental caries"[MeSH Terms] OR ("dental"[All Fields] AND "caries"[All Fields]) OR "dental caries"[All Fields]) OR ("dentural"[All Fields] OR "denture s"[All Fields] OR "dentures"[MeSH Terms] OR "dentures"[All Fields] OR "denture"[All Fields]) OR (("mouth"[MeSH Terms] OR "mouth"[All Fields] OR "mouths"[All Fields] OR "mouth s"[All Fields] OR "mouthed"[All Fields] OR "mouthful"[All Fields] OR "mouthfuls"[All Fields] OR "mouthing"[All Fields]) AND "disease*"[All Fields]) OR "periodont*"[All Fields] OR ("mouth neoplasms"[MeSH Terms] OR ("mouth"[All Fields] AND "neoplasms"[All Fields]) OR "mouth neoplasms"[All Fields] OR ("oral"[All Fields] AND "cancer"[All Fields]) OR "oral cancer"[All Fields]) OR ("mouth neoplasms"[MeSH Terms] OR ("mouth"[All Fields] AND "neoplasms"[All Fields]) OR "mouth neoplasms"[All Fields] OR ("mouth"[All Fields] AND "cancer"[All Fields]) OR "mouth cancer"[All Fields]) OR (("mouth"[MeSH Terms] OR "mouth"[All Fields] OR "oral"[All Fields]) AND "tumor*"[All Fields]) OR (("mouth"[MeSH Terms] OR "mouth"[All Fields] OR "mouths"[All Fields] OR "mouth s"[All Fields] OR "mouthed"[All Fields] OR "mouthful"[All Fields] OR "mouthfuls"[All Fields] OR "mouthing"[All Fields]) AND "tumor*"[All Fields]) OR ("gingiva"[MeSH Terms] OR "gingiva"[All Fields] OR "gingival"[All Fields] OR "gingivally"[All Fields] OR "gingivals"[All Fields] OR "gingivitis"[MeSH Terms] OR "gingivitis"[All Fields] OR "gingivitides"[All Fields]) OR ("malocclusal"[All Fields] OR "malocclusion"[MeSH Terms] OR "malocclusion"[All Fields] OR "malocclusions"[All Fields] OR "malocclusive"[All Fields]) OR (("gingiva"[MeSH Terms] OR "gingiva"[All Fields] OR "gingival"[All Fields] OR "gingivally"[All Fields] OR "gingivals"[All Fields] OR "gingivitis"[MeSH Terms] OR "gingivitis"[All Fields] OR "gingivitides"[All Fields]) AND "disease*"[All Fields]) OR "gingivit*"[All Fields] OR "crossbite*"[All Fields] OR ("fluorosis, dental"[MeSH Terms] OR ("fluorosis"[All Fields] AND "dental"[All Fields]) OR "dental fluorosis"[All Fields] OR ("dental"[All Fields] AND "fluorosis"[All Fields])) OR (("teeth s"[All Fields] OR "teeths"[All Fields] OR "tooth"[MeSH Terms] OR "tooth"[All Fields] OR "teeth"[All Fields] OR "tooth s"[All Fields] OR "tooths"[All Fields]) AND ("mortality"[MeSH Terms] OR "mortality"[All Fields] OR "mortalities"[All Fields] OR "mortality"[MeSH Subheading])) OR ("tooth loss"[MeSH Terms] OR ("tooth"[All Fields] AND "loss"[All Fields]) OR "Edentulism"[All Fields] OR "Bruxism"[All Fields] OR "Prosthetic Need"[All Fields] "tooth loss"[All Fields]))) | 1128 |
| 2. | **Scopus:**  ((Oral Health) or (Mouth Diseases) or Tooth* or Teeth* or (Dental Caries) or Denture or (Mouth Disease*) or Periodont* or (Oral Cancer) or (Mouth Cancer) or (Oral Tumor*) or (Mouth Tumor*) or Gingivitis or Malocclusion or (Gingival Disease*) or Gingivit* or Crossbite* or (dental fluorosis) or (Tooth Mortality) or (tooth loss)) and ((Value of Life) or (Quality of Life) or (Quality-Adjusted Life Years) or DALY* or QALY* or (Quality-Adjusted Related Quality) or HRQOL or QoL or (Quality of Life) or (Dental Impact) or (Social Impact) or (Health Impact) or COHQOL or CHILD-OIDP or OQLQ or OHQoL or DIDL or ECOHIS or CPQ or OHIP or GOHAI) and India* | 323 |
| 3. | **CINAHL**  ("Oral Health" OR "Mouth disease" OR "Tooth" OR "Teeth" OR " Dental caries" OR "Gingivi*" OR "Gingival disease" OR "Periodontal disease" OR "Periodont*" OR "Malocclusion" OR "cross-bite" OR "dental fluorosis" OR "Tooth loss" OR "edentulism" OR "oral cancer" OR "Mouth tumor" OR "tooth mortality" OR "bruxism" OR "prosthetic*" )AND ("Quality of life" OR " oral health quality of life" OR "QoL" OR "Value of life" OR " Quality-adjusted life years" OR "DALY" OR " QALY" OR "HRQOL" OR "Dental impact" OR " Social impact" OR "Health impact" OR "COHQOL" OR "CHILD- OIDP" OR "OHQOL" OR "CPQ" OR "OHIP" OR "GOHAI" OR "ECOHIS" OR "DIDL") AND (India*) | 135 |
| 4. | **Web of Science**  ("Oral Health" OR "Mouth disease" OR "Tooth" OR "Teeth" OR " Dental caries" OR "Gingivi*" OR "Gingival disease" OR "Periodontal disease" OR "Periodont*" OR "Malocclusion" OR "cross-bite" OR "dental fluorosis" OR "Tooth loss" OR "edentulism" OR "oral cancer" OR "Mouth tumor" OR "tooth mortality" OR "bruxism" OR "prosthetic*" )AND ("Quality of life" OR " oral health quality of life" OR "QoL" OR "Value of life" OR " Quality-adjusted life years" OR "DALY" OR " QALY" OR "HRQOL" OR "Dental impact" OR " Social impact" OR "Health impact" OR "COHQOL" OR "CHILD- OIDP" OR "OHQOL" OR "CPQ" OR "OHIP" OR "GOHAI" OR "ECOHIS" OR "DIDL") AND (India*) | 773 |
| 4 | **PsycInfo**  ("Oral Health" OR "Mouth disease" OR "Tooth" OR "Teeth" OR " Dental caries" OR "Gingivi*" OR "Gingival disease" OR "Periodontal disease" OR "Periodont*" OR "Malocclusion" OR "cross-bite" OR "dental fluorosis" OR "Tooth loss" OR "edentulism" OR "oral cancer" OR "Mouth tumor" OR "tooth mortality" OR "bruxism" OR "prosthetic*" )AND ("Quality of life" OR " oral health quality of life" OR "QoL" OR "Value of life" OR " Quality-adjusted life years" OR "DALY" OR " QALY" OR "HRQOL" OR "Dental impact" OR " Social impact" OR "Health impact" OR "COHQOL" OR "CHILD- OIDP" OR "OHQOL" OR "CPQ" OR "OHIP" OR "GOHAI" OR "ECOHIS" OR "DIDL") AND (India*) | 22 |
| 5. | **Google scholar**  Oral disease, India and Quality of life  Articles from first 20 pages considered | 75 |

**S2: Studies ineligible following full- text review**

1. Acharya S. Oral health-related quality of life and its associated factors in an Indian adult population. Oral Health Prev Dent. 2008;6:175–84.

Reason for exclusion: Quality of life of individuals with and without disease is not mentioned

1. Acharya S, Tandon S. The effect of early childhood caries on the quality of life of children and their parents. Contemp Clin Dent. 2011;2(2):98–101.

Reason for exclusion: Quality of life of individuals with and without disease is not mentioned

1. Agarwal SK, Munjal M, Koul R, Agarwal R. Prospective evaluation of the quality of life of oral tongue cancer patients before and after the treatment. Ann Palliat Med. 2014;3(4):238–43.

Reason for exclusion: Quality of life of individuals without disease is not mentioned

1. Baliah J, Keluskar V, Livingstone D, Panwar A. Evaluation of pain management and quality of life among oral cancer patients - A cross sectional study. J Indian Acad Oral Med Radiol. 2021;33(2):124-128.

Reason for exclusion: Assessed the association between pain and quality of life

1. Bandela V, Munisekhar MS, Patil SR, Nagarajappa AK, Farilqi S, Metta KK, Alam MK, Kanaparthi S. Oral Health-Related Quality of Life (OHRQoL) in Patients' with Dental Prosthesis. Pesquisa Brasileira em Odontopediatria e Clínica Integrada 2020; 20:e0006

Reason for exclusion: Not assessed Quality of life of individuals without disease

1. Banerjee S, Banerjee R, Shenoy U, Agarkar S, Bhattacharya S. Effect of orthodontic pain on quality of life of patients undergoing orthodontic treatment. Indian J Dent Res. 2018;29(1):4-9.

Reason for exclusion: Assessed the association between pain and quality of life

1. Bashir A, Kumar D, Dewan D, Sharma R. Quality of life of head and neck cancer patients before and after cancer-directed treatment – A longitudinal study. J Can Res Ther. 2020;16(3):500-507.

Reason for exclusion: Assessed the quality of life before and after treatment

1. Bhargava N, Jadhav A, Kumar P, Kapoor A, Mudrakola D, Singh S. Oral health-related quality of life and severity of periodontal disease. J Pharm Bioall Sci. 2021;13(5): S387-S390.

Reason for exclusion: Not assessed Quality of life of individuals without disease

1. Bhat M, Bhat S, Brondani M, Mejia GC, Pradhan A, Roberts-Thomson K, et al. Prevalence, Extent, and Severity of Oral Health Impacts Among Adults in Rural Karnataka, India. JDR Clinical & Translational Research. 2021 Apr;6(2):242–50.

Reason for exclusion: Quality of life of individuals with and without disease is not mentioned

1. Bhatia R, J. J, Mehta N. Impact of malocclusion on oral health‑related quality of life in 10–14‑year‑old children of Mumbai, India. Contemp Clin Dent. 2016;7:445-50.

Reason for exclusion: Quality of life of individuals with and without disease is not mentioned

1. Binnal A, Rajesh G, Saxena P, Banerjee S, Denny C, Tadakamadla SK. Health‐related quality of life among oral and oropharyngeal cancer patients: An exploratory study. Oral Diseases. 2022 Apr;28(3):585–99.

Reason for exclusion: Quality of life individuals without disease is not mentioned

1. Bramantoro T, Sari B, Ramadhan A. Relationship between Dental Caries Prevalence and Elderly’s Quality of Life. Indian J Public Health Res Dev. 2020;11(7):1514–9.

Reason for exclusion: Quality of life of individuals with and without disease is not mentioned

1. Freitas et al. Influence of two caries detection strategies on the quality of life of preschool children: An analysis of secondary outcomes of a 2-Year randomized clinical trial. Community Dent Oral Epidemiol. 2022 Jun 7. doi: 10.1111/cdoe.12765.

Reason for exclusion: Oral health status not mentioned

1. Chahar P, Mohanty VR, Aswini YB. Oral health-related quality of life among elderly patients visiting special clinics in public hospitals in Delhi, India: A cross-sectional study. Indian Journal of Public Health. 2019 Jan 1;63(1):15-20.

Reason for exclusion: Number of individuals with and without disease is not mentioned

1. Chakradhar K, Doshi D, Kulkarni S, Reddy BS, Reddy S, Srilatha A. Self perceived psychosocial impact of dental aesthetics among young adults: a cross sectional questionnaire study. International Journal of Adolescent Medicine and Health. 2020 Aug 18;32(3):1-8.

Reason for exclusion: Quality of life of individuals with and without disease is not mentioned

1. Chakravathy KP, Thippeswamy HM, Kumar N, Chenna D. Relationship of body mass index and dental caries with oral health related quality of life among adolescents of Udupi district, South India. Eur Arch Paediatr Dent. 2013 Jun;14(3):155–9.

Reason for exclusion: Quality of life of individuals with and without disease is not mentioned

1. Chaudhry K, Bali R, Patnana AK, Chattopadhyay C, Sharma PP, Khatana S. Impact of Oral Submucous Fibrosis on Quality of Life: A Multifactorial Assessment. J Maxillofac Oral Surg. 2020 Jun;19(2):251–6.

Reason for exclusion: Not assessed quality of life among individuals without disease

1. Chaudhry K, Bali R, Patnana AK, Bindra S, Jain G, Sharma PP. Impact of Oral Submucous Fibrosis on Quality of Life: A Cross-Sectional Study. J Maxillofac Oral Surg. 2019 Jun;18(2):260–5.

Reason for exclusion: Not assessed quality of life among individuals without disease

1. Chaukar DA, Walvekar RR, Das AK, Deshpande MS, Pai PS, Chaturvedi P, et al. Quality of life in head and neck cancer survivors: a cross-sectional survey. American Journal of Otolaryngology. 2009 May;30(3):176–80.

Reason for exclusion: Not assessed quality of life among individuals without disease

1. Chellappa, LR, Arumugham IM, Srisakthi D. Oral health related quality of life among institutionalized and non institutionalized elderly population in india- a structured review. 2020;17(7): 847-857.

Reason for exclusion: A review

1. Chole RH, Patil R. Assessment of the quality of life and performance status in patients with oral submucous fibrosis in central India. Medicine and Pharmacy Reports. 2018 Apr 26;91(2):203–8.

Reason for exclusion: Not assessed quality of life among individuals without disease

1. Dable RA, Nazirkar GS, Singh SB, Wasnik PB. Assessment of Oral Health Related Quality of Life Among Completely Edentulous Patients in Western India by Using GOHAI. J Clin Diagn Res Sep 2013;7(9):2063-7

Reason for exclusion: Assessed the effect of treatment on Quality of Life

1. Deb Barma M, Indiran MA, Kumar R P, Balasubramaniam A, Kumar MPS. Quality of life among head and neck cancer treated patients in South India: A cross-sectional study. Journal of Oral Biology and Craniofacial Research. 2021 Apr;11(2):215–8.

Reason for exclusion: Assessed the effect of treatment on Quality of Life

1. Deep A, Singh M, Sharma R, Singh M, Mattoo K. Perceived oral health status and treatment needs of dental students. Natl J Maxillofac Surg. 2020;11(1):76-80.

Reason for exclusion: Not assessed the oral health status of the individuals

1. Sanchana, V B, Dr. Suresh. Oral health related quality of life for patients treated with complete dentures, removable and fixed partial dentures. International journal of recent advances in multidisciplinary research. 2015;2(7):0526–9.

Reason for exclusion: Assessed effect of treatment on Quality of Life

1. Deshmukh SP, Radke UM. Retracted: Translation and validation of the Hindi version of the Geriatric Oral Health Assessment Index: Translation and validation of the Hindi version. Gerodontology. 2012 Jun;29(2):e1052–8.

Reason for exclusion: Translation and validation of Quality of Life assessment tools (Article retracted)

1. Dhama K, Razdan P, Niraj L, Ali I, Patthi B, Kundra G. Magnifying the senescence: Impact of oral health on quality of life and daily performance in geriatrics: A cross-sectional study. J Int Soc Prevent Communit Dent. 2017;7(8): S113-S118.

Reason for exclusion: Number of individuals with and without disease is not mentioned

1. Dharsini S, Ramakrishnan M, Ganapathy D. The effect of severe caries on the quality of life in young children: A cross-sectional study. Drug Invention Today. 2020;14(3):498-501.

Reason for exclusion: Not assessed Quality of Life of individuals without disease

1. Dhawan P, Singh A, Agarwal A, Aeran H. Psychometric properties of Hindi version of child oral impact on daily performances (C-OIDP) index amongst school children in North India. Journal of Oral Biology and Craniofacial Research. 2019 Jan;9(1):10–3.

Reason for exclusion: Translation and validation of Quality of Life assessment tools

1. Dhingra S, Rajesh G, Rao A, Pai U, Shenoy R, Pai M. Impact of occlusal support and perceived chewing ability on oral health-related quality of life among patients attending a private dental institution in India. J Indian Prosthodont Soc. 017;17(1):15-21

Reason for exclusion: Oral health status not assessed

1. Dholam K, Chouksey G, Dugad J. Oral health-related quality of life after prosthetic rehabilitation in patients with oral cancer: A longitudinal study with the Liverpool Oral Rehabilitation Questionnaire version 3 and Oral Health Impact Profile-14 questionnaire. Indian J Cancer. 2016;53(2):256-260.

Reason for exclusion: Assessed the effect of treatment on Quality of life

1. Fotedar S, Chauhan A, Bhardwaj V, Manchanda K, Fotedar V. Association between oral health status and oral health-related quality of life among the prison inmate population of kanda model jail, Shimla, Himachal Pradesh, India. Indian J Public Health. 2016;60(2):150-3.

Reason for exclusion: Quality of life of individuals with and without disease is not mentioned

1. Fotedar S, Sharma KR, Fotedar V, Bhardwaj V, Chauhan A, Manchanda K. Relationship between Oral Health Status and Oral Health Related Quality Of Life in Adults Attending H.P Government Dental College, Shimla, Himachal Pradesh- India. 2014;13(3): 661-5.

Reason for exclusion: Quality of life of individuals with and without disease is not mentioned

1. Gandhi AK, Roy S, Thakar A, Mohanti BK. Symptom Burden and Quality of Life in Advanced Head and Neck Cancer Patients: AIIMS Study of 100 Patients. Indian J Palliat Care. 2014;20(3):89-193.

Reason for exclusion: Quality of life of individuals with and without disease is not mentioned

1. Ganesh G, Tripathi T, Rai P. Effect of fixed mechanotherapy on psychosocial impact of malocclusion using PIDAQ in the Indian population - A follow up study. J Oral Biol Craniofac Res Jan-Mar 2021;11(1):92-98.

Reason for exclusion: Assessed effect of treatment on Quality of Life

1. Garg K. Prospective Evaluation of Psychosocial Impact after One Year of Orthodontic Treatment Using PIDAQ Adapted for Indian Population. J. Clin. Diagnostic Res. 2017, Vol-11(8): ZC44-ZC48

Reason for exclusion: Assessed effect of treatment on Quality of Life

1. Geetha Priya P, Asokan S, Kandaswamy D. Objective and subjective parameters of oral health in South Indian children: A cross-sectional study. Indian J Dent Res. 2018;29(2):161-165.

Reason for exclusion: Quality of life of individuals with and without disease is not mentioned

1. Geevarghese A, Baskaradoss JK, Sarma PS. Oral Health-Related Quality of Life and Periodontal Status of Pregnant Women. Matern Child Health J. 2017 Aug;21(8):1634–42.

Reason for exclusion: Not assessed Quality of Life of individuals without disease

1. Gondivkar S, Bhowate R, Gadbail A, Gaikwad R, Gondivkar R, Sarode S, et al. Development and validation of oral health-related quality of life measure in oral submucous fibrosis. Oral Dis. 2018 Sep;24(6):1020–8.

Reason for exclusion: Development and validation of Quality of Life assessment tools

1. Gondivkar SM, Bhowate RR, Gadbail AR, Gondivkar RS, Sarode SC, Saode GS. Comparison of generic and condition-specific oral health-related quality of life instruments in patients with oral submucous fibrosis. Qual Life Res. 2019 Aug;28(8):2281–8.

Reason for exclusion: Quality of life of individuals with and without disease is not mentioned

1. Gondivkar SM, Bhowate RR, Gadbail AR, Gondivkar RS, Sarode SC, Sarode GS, et al. Impact of oral submucous fibrosis on oral health-related quality of life: A condition-specific OHRQoL-OSF instrument analysis. Oral Diseases. 2018;24(8):1442–8.

Reason for exclusion: Not assessed quality of individuals without disease

1. Gondivkar RS, Bhowate RR, Gadbail AR, Sarode SC, Gondivkar RS, Yuwanati M, et al. Quality of Life-related “Patient-reported Outcome Measures” in Oral Submucous Fibrosis Patients. The Journal of Contemporary Dental Practice. 2018 Mar;19(3):331–8.

Reason for exclusion: Not assessed Quality of Life of individuals without disease

1. Gondivkar SM, Gadbail AR, Gondivkar RS, Sarode SC, Sarode GS, Patil S. Impact of oral potentially malignant disorders on quality of life: a systematic review. Future Oncology. 2018 Apr;14(10):995–1010.

Reason for exclusion: A systematic Review

1. Gondivkar S, Gadbail A, Sarode S, Dasgupta S, Sharma B, Hedaoo A, et al. Prevalence of Trismus and Its Impact on Oral Health-Related Quality of Life in Patients Treated for Oral Squamous Cell Carcinoma. Asian Pac J Cancer Prev. 2021 Aug 1;22(8):2437–44.

Reason for exclusion: Quality of life individuals without disease is not assessed

1. Gondivkar SM, Gadbail AR, Sarode SC, Gondivkar RS, Yuwanati M, Sarode GS, et al. Measurement properties of oral health related patient reported outcome measures in patients with oral cancer: A systematic review using COSMIN checklist. PLoS One 2019;14(6):e0218833

Reason for exclusion: A systematic Review

1. Gondivkar SM, Gadbail AR, Sarode SC, Hedaoo A, Dasgupta S, Sharma B, et al. Oral and general health-related quality of life in oral squamous cell carcinoma patients- comparative analysis of different treatment regims. Journal of Oral Biology and Craniofacial Research. 2021 Apr;11(2):125–31.

Reason for exclusion: Assessed effect of treatment on Quality of Life

1. Grover V, Malhotra R, Dhawan S, Kaur G. Comparative Assessment of Oral Health Related Quality of Life in Chronic Periodontitis Patients of Rural and Urban Populations in Punjab. Oral Health and Preventive Dentistry. 2016 Jun 3;14(3):235–40.

Reason for exclusion: Not assessed Quality of Life of individuals without disease

1. Gupta DA, Ankola DAV, Hebbal DM. Prevalence, Intensity and Extent of Oral Impact on Daily Performances and their relationship to Oral health problems among rural females. Public Health. 2012;4(5):419-477.

Reason for exclusion: Number of individuals with and without disease is not mentioned

1. Gupta T, Sadana G, Rai HK. Effect of Esthetic Defects in Anterior Teeth on the Emotional and Social Well-being of Children: A Survey. International Journal of Clinical Pediatric Dentistry. 2019 Jun;12(3):229–32.

Reason for exclusion: Quality of life of individuals with and without disease is not mentioned

1. Indrapriyadharshini K, Madankumar P, Karthikeyan G. Oral health-related quality of life in patients treated for oral malignancy at Kanchipuram district, India: A cross-sectional study. Indian J Cancer. 2017;54:11–5.

Reason for exclusion: Assessed effect of treatment on Quality of Life

1. Janapareddy K, Parlapalli V, Pydi S, Pottem N, Chatti P, Pallekonda AP. Oral Health Status and Oral Health-Related Quality of Life (OHRQoL) among steel factory workers of Visakhapatnam-A cross-sectional study. J Family Med Prim Care. 2020;9(10):5309-5315.

Reason for exclusion: Quality of life of individuals with and without disease is not mentioned

1. Jena AK, Rautray S, Mohapatra M, Singh S. Oral health-related quality of life among male subjects with oral submucous fibrosis in a tertiary care hospital. Indian J Public Health. 2018;62(4):271–6.

Reason for exclusion: Not assessed Quality of life of individuals without disease

1. Jha K. Prevalence of Malocclusion and its Psycho-Social Impact among 12 To 15-Year-old School Children in Lucknow City. J Clin Diagn Res Oct 2014;8(10):ZC36-9

Reason for exclusion: Quality of life of individuals with and without disease is not mentioned

1. Johnson JE.,Varma PR, Maheswari E, Saraswathy GR. Assessment of health and quality of life of children residing in selected shelter homes in Bangalore. J. Appl. Pharm. Sci. 2018;8(4):84-89.

Reason for exclusion: Not assessed Quality of Life of individuals without disease

1. Kamatchinathan P, Kaja N, Muthuraman V, Antharaju Y, Kumar M, Varadharajan U. Psychological Analysis of Oral Cancer Patients during Pre-operative Period in South Indian Population: A Prospective, Quantitative, Multicentre Study. J Clin Diagn Res Oct 2016;10(10):ZC72-ZC74

Reason for exclusion: Not assessed Quality of Life of individuals without disease

1. Kapur N, Singla N, Kudva A, John ER. Evaluation of quality of life in patients surgically treated for potentially malignant oral lesions. Indian J Cancer Dec 2020:1-7

Reason for exclusion: Not assessed Quality of Life of individuals without disease

1. Kaur P, Singh S, Mathur A, Makkar DK, Aggarwal VP, Batra M, Sharma A, Goyal N. Impact of Dental Disorders and its Influence on Self Esteem Levels among Adolescents. J Clin Diagn Res Apr 2017;11(4):ZC05-ZC08

Reason for exclusion: Quality of life of individuals with and without disease is not mentioned

1. Khandelwal A, Neeli A, Gadiyar A. Assessment of quality of life of patients 1-5 years after treatment for oral cancer. Indian J Dent Res Sep-Oct 2017;28(5):538-544.

Reason for exclusion: Assessed the effect of treatment on Quality of life

1. Krittika R, Ramakrishnan M. Development and Validation of Tamil Version of Ecohis Scale T Ecohis: A Cross Sectional Study. JOURNAL OF RESEARCH IN MEDICAL AND DENTAL SCIENCE 2021;9(2):187-190

Reason for exclusion: Not assessed quality of life of individuals without disease

1. Kumar A, Puranik MP, Sowmya KR, Rajput S. Impact of occupational dental erosion on oral health-related quality of life among battery factory workers in Bengaluru, India. Dent Res J (Isfahan) Jan-Feb 2019;16(1):12-17.

Reason for exclusion: Quality of life of individuals with and without disease is not mentioned

1. Kumar A, Puranik MP, Sowmya KR, Rajput S. Impact of occupational dental erosion on oral health-related quality of life among battery factory workers in Bengaluru, India. Dent Res J (Isfahan) Jan-Feb 2019;16(1):12-17.

Reason for exclusion: Quality of life of individuals with and without disease is not mentioned

1. Kumar K, Khandpur M, Khandpur S, Mehrotra D, Chandra Tiwari S, Kumar S. Quality of life among Oral Potentially Malignant Disorder (OPMD) patients: A prospective study. J Oral Biol Craniofac Res Jan-Mar 2021;11(1):88-91

Reason for exclusion: Quality of life of individuals without disease is not mentioned

1. Kumar S, Badiyani, BK, Kumar A, Dixit, G, Sharma P, Agrawal S. Orofacial pain and quality of life in early adolescents in India. Int. J. Adolesc. Med. Health 2018;30(2).

Reason for exclusion: Assessed pain and Quality of Life

1. Kundapur V, Hegde R, Shetty M, Mankar S, Hilal M, Prasad A H. Effect of Loss of Teeth and its Association with General Quality of Life using Geriatric Oral Health Assessment Index (Gohai) among Older Individuals Residing in Rural Areas. Int J Biomed Sci Mar 2017;13(1):6-12.

Reason for exclusion: Oral health status not assessed

1. Lakshmi KPD, Aswath Narayanan MB, Ramesh Kumar SG, Selvamary AL, Sujatha A. Permanent Anterior Teeth Fractures and its Impact on Oral Health-Related Quality of Life among 8-15-Year-Old Schoolchildren of Chennai City - A Cross-Sectional Survey. Int J Appl Basic Med Res Jan-Mar 2020;10(1):30-36.

Reason for exclusion: Quality of life of individuals with and without disease is not mentioned

1. M R, Sen M, Mala K, Sujir N, Poojary D, Shetty NJ, Shenoy R, Saldanha S. Critical Assessment on Unmet Oral Health Needs and Oral Health-related Quality of Life Among Old Age Home Inhabitants in Karnataka, India. Clin Cosmet Investig Dent 2021;13():181-186.

Reason for exclusion: Not assessed Quality of life of individuals without disease

1. Machale P, Hegde-Shetiya S, Shirahatti, Agarwal, D. Oral health related quality of life (OHRQoL) amongst patients wearing fixed orthodontic appliance in Pimpri, Pune, India - A cross sectional study. Pesqui. Bras. Odontopediatria Clin. Integr. 2012;12(3):351-356.

Reason for exclusion: Not assessed Quality of life of individuals without disease

1. Mandava P, Singaraju GS, Obili S, Nettam V, Vatturu S, Erugu S. Impact of self-esteem on the relationship between orthodontic treatment and the oral health-related quality of life in patients after orthodontic treatment - a systematic review. Med Pharm Rep Apr 2021;94(2):158-169.

Reason for exclusion: A systematic review

1. Mansoori S, Mehta A, Ansari MI. Factors associated with Oral Health Related Quality of Life of children with severe -Early Childhood Caries. J Oral Biol Craniofac Res Jul-Sep 2019;9(3):222-22.

Reason for exclusion: Not assessed Quality of life of individuals without disease

1. Martha L, Bhaduri A, Jain AG. Impact of oral cancer & related factors on the quality of life (QOL) of patients. Nurs J India Jun 2004;95(6):129-31.

Reason for exclusion: Not assessed Quality of life of individuals without disease

1. Martins, Milene T, Sardenberg, Fernanda, Bendo, Cristiane B, Abreu, Mauro Henrique, Vale, Míriam P, Paiva, Saul M; Pordeus, Isabela A. Dental caries remains as the main oral condition with the greatest impact on children’s quality of life. PLoS One 2017;12(10) :e0185365

Reason for exclusion: Quality of life of individuals with and without disease is not mentioned

1. Mary AV, Mahendra J, John J, Moses J, Ebenezar AVR, Kesavan R. Assessing Quality of Life using the Oral Health Impact Profile (OHIP-14) in Subjects with and without Orthodontic Treatment need in Chennai, Tamil Nadu, India. J Clin Diagn Res Aug 2017;11(8):ZC78-ZC81.

Reason for exclusion: Quality of life is expressed in mean Rank

1. Marya, Charu Mohan, Baiju, CS, Nagpal, Ruchi, Rekhi, Amit. Oral Health Related Quality of Life And It's Impact on Elderly People. Indian Journal of Dental Sciences 2012;4.

Reason for exclusion: Quality of life of individuals with and without disease is not mentioned

1. Mathur VP, Dhillon JK, Logani A, Agarwal R. Development and validation of oral health-related early childhood quality of life tool for North Indian preschool children. Indian J Dent Res Sep-Oct 2014;25(5):559-66.

Reason for exclusion: Translation and validation of Quality of life tools

1. . Mathur VP, Jain V, Pillai RS, Kalra S. Translation and validation of Hindi version of Geriatric Oral Health Assessment Index. Gerodontology Mar 2016;33(1):89-96.

Reason for exclusion: Translation and validation of Quality of life tools

1. Mehta, Abhishek, Govind, Murali, Broadbent, Jonathan. Oral health-related quality of life of older patients attending a government dental hospital in India. Journal of Indian Association of Public Health Dentistry 2020;18(2):151-155.

Reason for exclusion: Quality of life of individuals with and without disease is not mentioned

1. Mukharjee S, Kumar V, Gupta S, Barua P, Prakash P, Das S, Akhtar N, Rajan S, Chaturvedi A. Quality of Life in Patients with Recurrent Oral Squamous Cell Carcinoma: A Study from India. J. Maxillofac. Oral Surg. 2021;():

Reason for exclusion: Not assessed quality of life individuals without disease

1. Muralidharan S, Acharya A, Koshy AV, Koshy JA, Yogesh TL, Khire B. Dentition status and treatment needs and its correlation with oral health-related quality of life among men having sex with men and transgenders in Pune city: A cross-sectional study. J Oral Maxillofac Pathol Sep-Dec 2018;22(3):443.

Reason for exclusion: Not assessed quality of life individuals without disease

1. Nagappan N, Madhanmohan R, Gopinathan NM, Stephen SR, Pillai DDM, Tirupati N. Oral Health-Related Quality of Life and Dental Caries Status in Children With Orofacial Cleft: An Indian Outlook. J Pharm Bioallied Sci May 2019;11(Suppl 2):S169-S174.

Reason for exclusion: Not assessed quality of life individuals without disease

1. Nagarajan S, Chandra R. Perception of oral health related quality of life (OHQoL-UK) among periodontal risk patients before and after periodontal. Community Dent Health 2012;29():90-4.

Reason for exclusion: Assessed effect of treatment on Quality of life

1. Nagarajappa R, Batra M, Sanadhya S, Daryani H, Ramesh G. Oral impacts on daily performance: Validity, reliability and prevalence estimates among Indian adolescents. Int J Dent Hyg May 2018;16(2):279-285.

Reason for exclusion: Oral health status not assessed

1. Nagarajappa R, Ramesh G, Sandesh N, Lingesha RT, Hussain MA. Impact of fixed orthodontic appliances on quality of life among adolescents' in India. J Clin Exp Dent Oct 2014;6(4):e389-94.

Reason for exclusion: Quality of life of individuals with and without disease is not mentioned

1. Naidu G, Shukla S, Nagi R, Jain S, Makkad R. Evaluation of oral health related quality of life in subjects diagnosed with head and neck malignancies undergoing chemotherapy, radiotherapy, and surgery. J. Indian Acad. Oral Med. Radiol. 2019;31(3):228-233.

Reason for exclusion: Assessed the effect of treatment on Quality of Life

1. Nayak SG, Pai MS, George LS. Quality of life of patients with head and neck cancer: A mixed method study. J Cancer Res Ther Jul-Sep 2019;15(3):638-644.

Reason for exclusion: Not assessed the Quality of Life among individuals without disease

1. Nirmala E, Remani KN, Stephen N. Environmentally mediated health impacts among the tribals of Attappady and Wayanad in the Wester Ghats region of Kerala. Asian J. Microbiol. Biotechnol. Environ. Sci. 2004;6(2):237-243.

Reason for exclusion: Quality of life is not assessed

1. Oberoi SS, Hiremath SS, Yashoda R, Marya C, Rekhi A. Prevalence of Various Orofacial Pain Symptoms and Their Overall Impact on Quality of Life in a Tertiary Care Hospital in India. J Maxillofac Oral Surg Dec 2014;13(4):533-8.

Reason for exclusion: Oral health status not assessed

1. Patanapu SK, Doshi D, Kulkarni S, Reddy BS, Srilatha A, Narayana DS. Correlation of oral health related quality of life with dentition status and treatment need among 12 year old school children of Dilsukhnagar, Hyderabad. Indian J Dent Res May-Jun 2020;31(3):343-349.

Reason for exclusion: Quality of life of individuals with and without disease is not mentioned

1. Patro SK, Panda NK, Bakshi J, Verma RK, Kumar P, Gaba S, John JR. Quality of Life in Patients with Reconstructions After Resections for Oral Cavity Cancers. Indian J Otolaryngol Head Neck Surg Oct 2019;71(Suppl 1):291-300.

Reason for exclusion: Assessed the effect of treatment on Quality of Life

1. Pattanaik S, Sahoo SN, Nanda SB, Nayak TK. Evaluation of quality of life in patients undergoing orthodontic treatment in eastern India population. Indian J. Public Health Res. Dev. 2018;9(11):1093-1097.

Reason for exclusion: Not assessed Quality of life of individuals without disease

1. Pavithran VK, Murali R, Krishna M, Shamala A, Yalamalli M, Kumar AV, Raina R. Impact of oral diseases on daily activities among 12- to 15-year-old institutionalized orphan and non-orphan children in Bengaluru city: A cross-sectional analytical study. Indian J Dent Res May-Jun 2020;31(3):396-402.

Reason for exclusion: Quality of life of individuals with and without disease is not mentioned

1. Pentapati KC, Acharya S, Bhat M, Krishna Rao SV, Singh S. Oral health impact, dental caries, and oral health behaviors among the National Cadets Corps in South India. J Investig Clin Dent Feb 2013;4(1):39-43.

Reason for exclusion: Quality of life of individuals with and without disease is not mentioned

1. Peter E, Baiju RM, Shivaraman R, Varghese NO, Varughese JM. Malocclusion-Related Quality of Life Questionnaire (MRQoLQ): Development and validation of a new psychometric tool for older adolescents with malocclusion. Dental Press J Orthod Nov-Dec 2019;24(6):28-35.

Reason for exclusion: Translation and validation of Quality of life tools

1. Piassi E, Antunes LS, Antunes LA. Orthodontic treatment reduces the impact on children and adolescents' oral health-related quality of life. Indian J Dent Res Mar-Apr 2016;27(2):213-9.

Reason for exclusion: A review

1. Pingili S, Ahmed J, Sujir N, Shenoy N, Ongole R. Evaluation of Malnutrition and Quality of Life in Patients Treated for Oral and Oropharyngeal Cancer. Scientific WorldJ ournal 2021;2021():9936715.

Reason for exclusion: Quality of life of individuals with and without disease is not mentioned

1. Praveen BH, Prathibha B, Reddy PP, Monica M, Samba A, Rajesh R .Co Relation between PUFA Index and Oral Health Related Quality of Life of a Rural Population in India: A Cross-Sectional Study. J Clin Diagn Res Jan 2015;9(1):ZC39-42.

Reason for exclusion: Quality of life of individuals with and without disease is not mentioned

1. Priya H, Sequeira PS, Acharya S, Kumar M. Oral health related quality of life among dental students in a private dental institution in India. J Int Soc Prev Community Dent Jul 2011;1(2):65-70.

Reason for exclusion: Oral health status not assessed

1. Priyadharshini S, Leelavathi L. Assessment of oral health related quality of life in patients with malocclusion. INTERNATIONAL JOURNAL OF EARLY CHILDHOOD SPECIAL EDUCATION 2022;14(2):955-964.

Reason for exclusion: Not assessed quality of life of individuals without disease

1. Purohit BM, Singh A, Acharya S, Bhat M, Priya H. Assessment and validation of the oral impact on daily performance (OIDP) instrument among adults in Karnataka, South India. Community Dent Health Sep 2012;29(3):203-8.

Reason for exclusion: Translation and validation of Quality of life tools

1. Punam M, Sonia M. Impact of oral health related quality of life in elderly with and without dentures. **J.** **Health Psychol**. 2011; 5(2):97-104.

Reason for exclusion: Assessed the effect of treatment

1. Ranasinghe an, Leelavathi l. Assessment of oral health quality of life among elderly population with edentulous jaws. International journal of early childhood special education 2022;14(2):618-628.

Reason for exclusion: Not assessed quality of life of individuals without disease

1. Ravi P, Rao A, Rajesh G, Shenoy R, Pai B.H.M. Relationship between geriatric oral health assessment index (GOHAI) and oral health status of the institutionalized elderly in Mangalore, India. Indian J. Public Health Res. Dev. 2019;10(4):146-151.

Reason for exclusion: Quality of life of individuals with and without disease is not mentioned

1. Routh S, Mithun Pai B.H, Rajesh G, Shenoy R, Sarit S. Relationship between dental anxiety, oral health related quality of life and oral health status of indian coast guard personnel in Mangalore, Karnataka. Indian J. Public Health Res. Dev. 2019;10(8):133-138.

Reason for exclusion: Oral health status not assessed

1. Rudraswamy, Sushma, Vijai S, Doggalli, Nagabhushana, Manjunath, Maurya, Siddana, Sunitha. Evaluation of Child Perceptions Questionnaire on Oral Health Related Quality of Life among School Children with Various Orofacial Conditions in Mysore, India. Asian Journal of Basic Science & Research 2020;2(3):23-27.

Reason for exclusion: Quality of life individuals without disease is not assessed

1. Saimadhavi N, Raju MAKV, Reddy, R Sudhakara, Ramesh T, Tabassum, D Ayesha, Ramya K. Impact of oral diseases on quality of life in subjects attending out-patient department of a dental hospital, India. ournal of Orofacial Sciences 2013;5(1):27.

Reason for exclusion: Quality of life individuals without disease is not assessed

1. Sakthivel P, K Irugu DV, Singh CA, Verma H, Yogal R, Jat B, Chadran A, Sikka K, Thakar A, Sharma SC . Quality of life outcome measures using University of Washington questionnaire version 4 in early T1/T2 anterior tongue cancers with and without radiotherapy: A cross-sectional study. Indian J Cancer Apr-Jun 2017;54(2):447-452.

Reason for exclusion: Quality of life of lndividuals without disease is not assessed

1. Samuel SR, Kuduruthullah S, Khair AMB, Al Shayeb M, Elkaseh A, Varma SR, Nadeem G, Elkhader IA, Ashekhi A. Impact of pain, psychological-distress, SARS-CoV2 fear on adults' OHRQOL during COVID-19 pandemic. Saudi J Biol Sci Jan 2021;28(1):492-494.

Reason for exclusion: Quality of life of individuals with and without disease is not mentioned

1. Samuel SR, Kuduruthullah S, Khair AMB, Shayeb MA, Elkaseh A, Varma SR. Dental pain, parental SARS-CoV-2 fear and distress on quality of life of 2 to 6 year-old children during COVID-19. Int J Paediatr Dent May 2021;31(3):436-441.

Reason for exclusion: Quality of life of individuals with and without disease is not mentioned

1. Satheeshkumar PS, Mohan MP. Necessitating a quality of life instrument specific to oral precancers/oral potentially malignant disorders. Oral Oncol Oct 2014;50(10):e51.

Reason for exclusion: Quality of life of individuals with and without disease is not mentioned

1. Sawai DS, Abdul NS, Rahman GS, Tabassum N, Kumar AP, Priyadarshni P. Mothers' sense of coherence and oral health-related quality of life in cleft lip and palate children visiting a private dental college: A Survey. J Family Med Prim Care Aug 2020;9(8):4386-4390.

Reason for exclusion: Oral health status not assessed

1. Saxena A, Nagarajappa R, Naik D, Abid M, Ramesh G. Assessing the effect of oral diseases on oral health related quality of life of institutionalized elderly using Oral Health Impact Profile (OHIP-14) questionnaire: a pilot study. Rocz Panstw Zakl Hig 2020;71(3):349-353

Reason for exclusion: Quality of life of individuals with and without disease is not mentioned

1. Shah AF, Batra M, Qureshi A. Evaluation of Impact of Pregnancy on Oral Health Status and Oral Health Related Quality of Life among Women of Kashmir Valley. J Clin Diagn Res May 2017;11(5):ZC01-ZC04.

Reason for exclusion: Quality of life of individuals with and without disease is not mentioned

1. Shaheen, S Sabiha, Kulkarni, Suhas, Doshi, Dolar, Reddy, Srikanth, Reddy, Padma. Oral health status and treatment need among institutionalized elderly in India. Indian Journal of Dental Research 2015;26(5):493.

Reason for exclusion: Quality of life of individuals with and without disease is not mentioned

1. Sharma, Rama, Hiregoudar, Mahesh, Prashant, GM, PG, Naveen Kumar, Chandu, GN. Oral health related quality of life of b. Ed students in davangere city, india. J Odontol Res 2013;1(1):17-24.

Reason for exclusion: Oral health status not assessed

1. Sharma Y, Mishra G, Parikh V. Quality of Life in Head and Neck Cancer Patients. Indian J Otolaryngol Head Neck Surg Oct 2019;71(Suppl 1):927-932.

Reason for exclusion: Quality of life of individuals with and without disease is not mentioned

1. Shavi GR, Thakur B, Bhambal A, Jain S, Singh V, Shukla A. Oral Health Related Quality of Life in Patients of Head and Neck Cancer Attending Cancer Hospital of Bhopal City, India. J Int Oral Health Aug 2015;7(8):21-7.

Reason for exclusion: Quality of life of individuals with and without disease is not mentioned

1. Shokry, Abd–Allah Eman, Adel, Mohammed Rehab, Rashad, Abo El-seoud Amany. Educational program to improve quality of life among elderly regarding oral health. Future Dental Journal 2018;4(2):211-215.

Reason for exclusion: Oral health status not assessed

1. Singh A, Agarwal A, Aeran H, Dhawan P. Oral Health & Quality of Life in preadolescents with hearing impairment in Uttarakhand, India. J Oral Biol Craniofac Res Apr-Jun 2019;9(2):161-165.

Reason for exclusion: Quality of life of individuals with and without disease is not mentioned

1. Singh A, Dhawan P, Gaurav V, Rastogi P, Singh S. Assessment of oral health-related quality of life in 9-15 year old children with visual impairment in Uttarakhand, India. Dent Res J (Isfahan) Jan-Feb 2017;14(1):43-49.

Reason for exclusion: Quality of life of individuals with and without disease is not mentioned

1. Singh AK, Kushwaha JK, Anand A, Sonkar AA, Husain N, Srivastava K, Singh S. Human Papilloma Virus in Oral Cavity Cancer and Relation to Change in Quality of Life Following Treatment-a Pilot Study from Northern India. Indian J Surg Oncol Dec 2016;7(4):386-391.

Reason for exclusion: Quality of life of individuals with and without disease is not mentioned

1. Singh, Saurabh, Saha, Sabyasachi, Singh, Sanjay, Shukla, Neha, Reddy, Vamsi K. Oral health-related quality of life among 12–15-year children suffering from dental fluorosis residing at endemic fluoride belt of Uttar Pradesh, India. Journal of Indian Association of Public Health Dentistry 2018;16(1):54

Reason for exclusion: Number of individuals with and without disease is not mentioned

1. Sinha N, Shankar D, Vaibhav V, Vyas T, Singh A, Parihar AS. Oral Health-Related Quality of Life in Children and Adolescents of Indian population. J Pharm Bioallied Sci Aug 2020;12(Suppl 1):S619-S622.

Reason for exclusion: Quality of life of individuals with and without disease is not mentioned

1. Sirisha NR, Srinivas P, Suresh S, Devaki T, Srinivas R, Simha, B Vikram. Oral health related quality of life among special community adult population with low socioeconomic status residing in Guntur city, Andhra Pradesh: A cross-sectional study. Journal of Indian Association of Public Health Dentistry 2014;12(4):302

Reason for exclusion: Number of individuals with and without disease is not mentioned

1. Subramaniam P; Surendran R. Oral Health Related Quality of Life and its Association with Dental Caries of Preschool Children in Urban and Rural Areas of India. J Clin Pediatr Dent 2020;44(3):154-160.

Reason for exclusion: Number of individuals with and without disease is not mentioned

1. Suguna S, Gurunathan D. Quality of life of children with sleep bruxism. J Family Med Prim Care Jan 2020;9(1):332-336.

Reason for exclusion: Quality of life not assessed for individuals without disease

1. Sudeep C, Sequeira, Peter Simon, Jain, Jithesh. Oral health related quality of life among 12-15 year old children residing at orphanages in South India-A descriptive study. British Journal of Research 2014;1(10):53-62.

Reason for exclusion: Not assessed Quality of life of individuals without disease

1. Sundaram NS, Narendar R, Dineshkumar P, Ramesh SB, Gokulanathan S. Evaluation of oral health related quality of life in patient with mild periodontitis among young male population of Namakkal district. J Pharm Bioallied Sci Jun 2013;5(Suppl 1):S30-2.

Reason for exclusion: Not assessed Quality of life of individuals without disease

1. Tabassum SN, Tupalli AR, Cheruku SR, Abidullah M, Rajajee K, Hussain TA. The Impact of Early Childhood Caries on Oral Health-Related Quality of Life of Children and Caregivers Residing in Rural and Urban Areas of the Rangareddy District. J Med Life Apr-Jun 2020;13(2):249-254.

Reason for exclusion: Quality of life of individuals with and without disease is not mentioned

1. Tadakamadla J, Kumar S, Lalloo R, Gandhi Babu DB, Johnson NW. Impact of oral potentially malignant disorders on quality of life. J Oral Pathol Med Jan 2018;47(1):60-65.

Reason for exclusion: Not assessed Quality of life of individuals without disease

1. Tariq, Amynah. Oral Health Related Quality of Life with DMFT of Undergraduates and Graduates of Dow University of Health Sciences. JPDA 2018;27(04):187.

Reason for exclusion: Quality of life of individuals with and without disease is not mentioned

1. Tewari N, Rahul GM, Ravi M. Correspondence for the article titled: "Impact of uncomplicated traumatic dental injuries on the quality of life of children and adolescents: a systematic review and meta-analysis". BMC Oral Health Mar 2021;21(1):133.

Reason for exclusion: A review

1. Thanvi J, Bumb D. Impact of dental considerations on the quality of life of oral cancer patients. Indian J Med Paediatr Oncol Jan 2014;35(1):66-70.

Reason for exclusion: Not assessed Quality of life of individuals without disease

1. Ulfah, Siti Fitria, Marjianto, Agus. Dental caries and oral health related to quality of life of children with disabilities. Indian J Forensic Med Toxicol 2019;13():1739-44.

Reason for exclusion: Not assessed Quality of life of individuals without disease

1. Veeraboina N, Doshi D, Kulkarni S, Patanapu SK, Dantala SN, Srilatha A. Tooth loss and oral health-related quality of life among adult dental patients: A cross-sectional study. Indian J Dent Res Jan-Mar 2022;33(1):2-6.

Reason: Not assessed QoL of individuals without disease

1. Veluthattil AC, Sudha SP, Kandasamy S, Chakkalakkoombil SV. Effect of Hypofractionated, Palliative Radiotherapy on Quality of Life in Late-Stage Oral Cavity Cancer: A Prospective Clinical Trial. Indian J Palliat Care Jul-Sep 2019;25(3):383-390.

Reason for exclusion: Effect of treatment on Quality of life

1. Venkatesan A, V AS, Ramalingam S, Seenivasan MK, Narasimhan M. Evaluation of Oral Health Status Using the Geriatric Oral Health Assessment Index Among the Geriatric Population in India: A Pilot Study. Cureus Mar 2020;12(3):e7344.

Reason for exclusion: Oral health status not assessed

1. Verma S, Sharma H. Translation and validation of hindi version of oral health impact profile-14, a measure of oral health-related quality of life of geriatrics. Indian J Dent Res Mar-Apr 2019;30(2):180-184.

Reason for exclusion: Translation and validation of Quality of life tools

1. Vignesh U, Mehrotra D, Bhave SM, Singh PK. Quality of life after distraction osteogenesis in TMJ ankylosis patients. Oral Surg Oral Med Oral Pathol Oral Radiol Mar 2021;131(3):295-303.

Reason for exclusion: Assessed Quality of life before and after treatment

1. Vyas S, Nagarajappa S, Dasar PL, Mishra P. Linguistic adaptation and psychometric evaluation of original Oral Health Literacy-Adult Questionnaire (OHL-AQ). J Adv Med Educ Prof Oct 2016;4(4):163-169.

Reason for exclusion: Assessed oral health literacy

1. Yashoda R, Puranik MP. Oral health status and parental perception of child oral health related quality-of-life of children with autism in Bangalore, India. J Indian Soc Pedod Prev Dent Apr-Jun 2014;32(2):135-9.

Reason for exclusion: Quality of life of individuals with and without disease is not mentioned

1. Yu, Shu-Juan, Chen, Peng, Zhu, Guo-Xiong. Relationship between implantation of missing anterior teeth and oral health-related quality of life. Quality of Life Research 2013;22(7):1613-1620.

Reason for exclusion: Study not conducted in India

1. Yuwanati M, Gondivkar S, Sarode SC, Gadbail A, Desai A, Mhaske S, Pathak SK, N Khatib M. Oral health-related quality of life in oral cancer patients: systematic review and meta-analysis. Future Oncol Mar 2021;17(8):979-990.

Reason for exclusion: A review

1. Yuwanati M, Gondivkar S, Sarode SC, Gadbail A, Sarode GS, Patil S, Mhaske S. Impact of Oral Lichen Planus on Oral Health-Related Quality of Life: A Systematic Review and Meta-Analysis. Clin Pract May 2021;11(2):272-286.

Reason for exclusion: A review


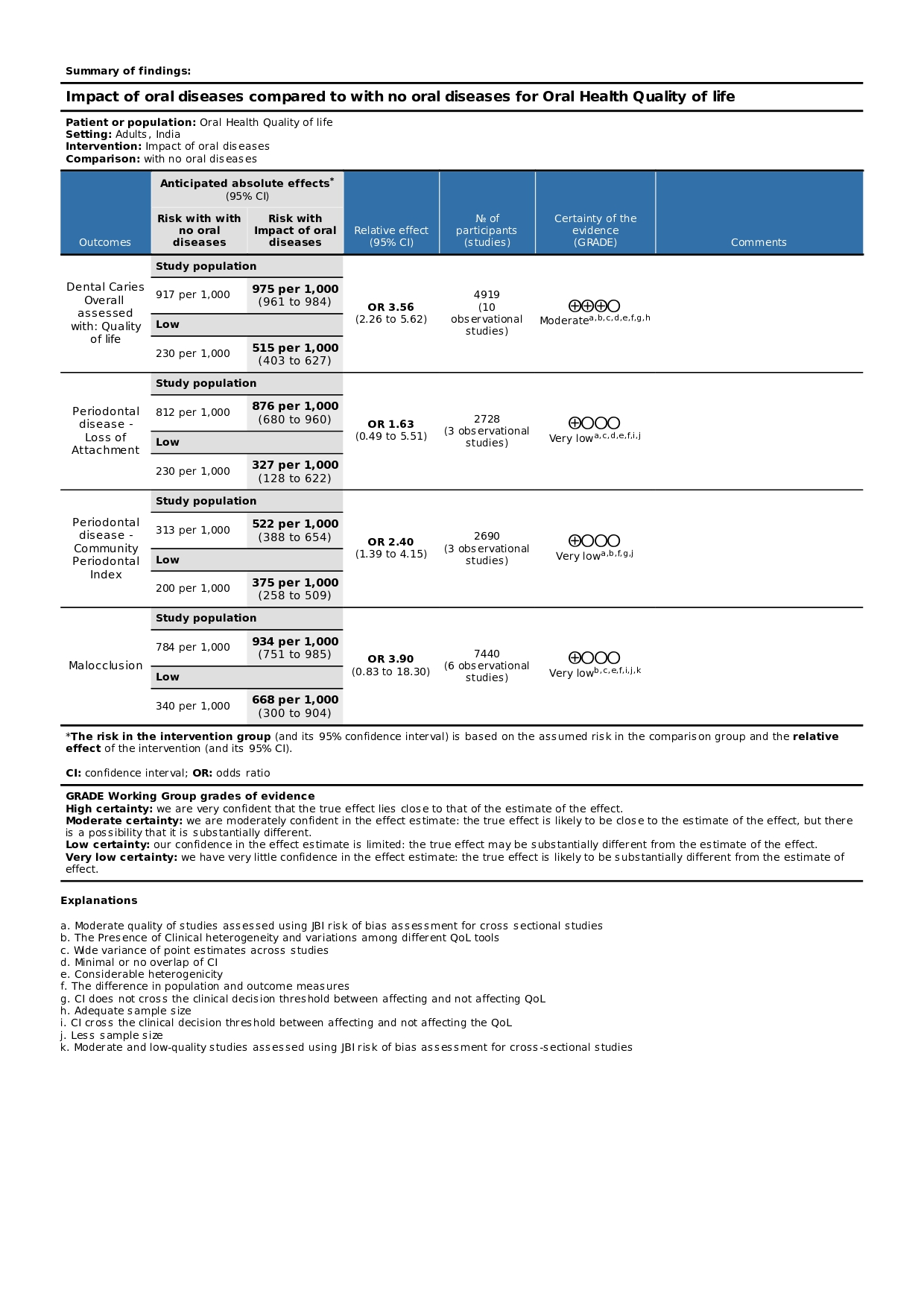


**S3- Summary of Findings (Dental caries, periodontal disease and malocclusion)**


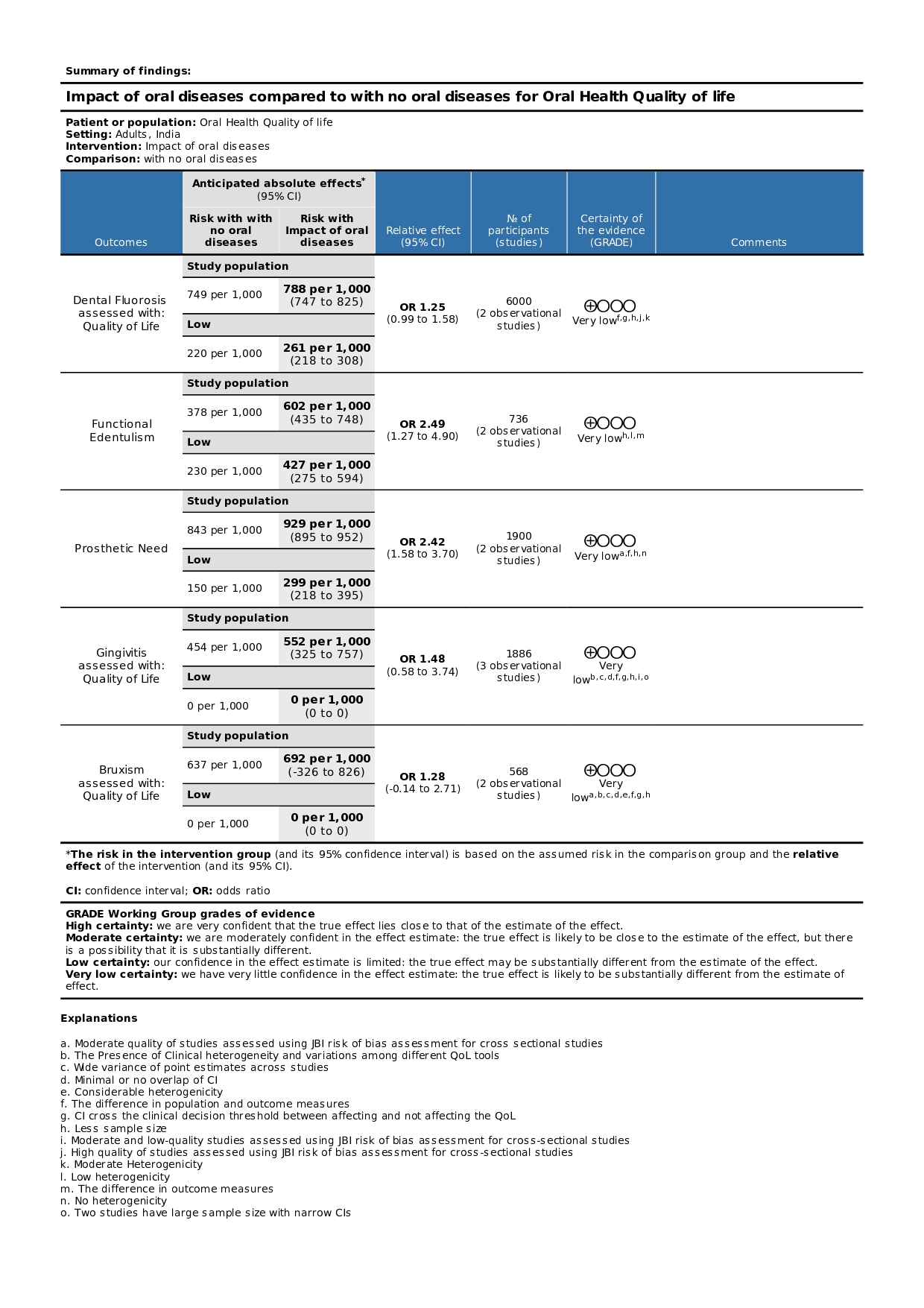


**S4- Summary of Findings (Dental fluorosis, functional edentulism, prosthetic need, gingivitis and bruxism)**


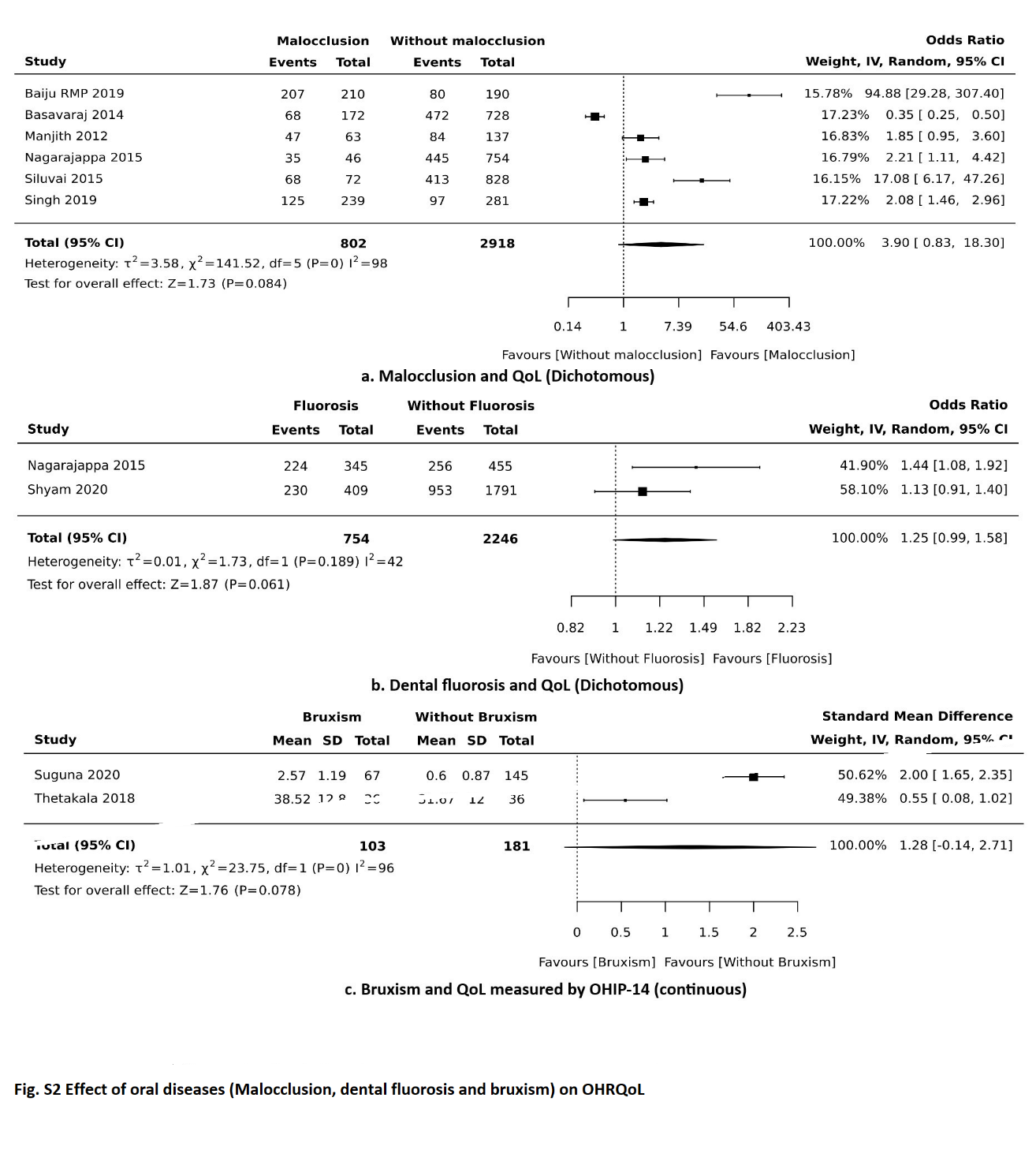


**S5- Effect of malocclusion, dental fluorosis, and bruxism on OHRQoL**


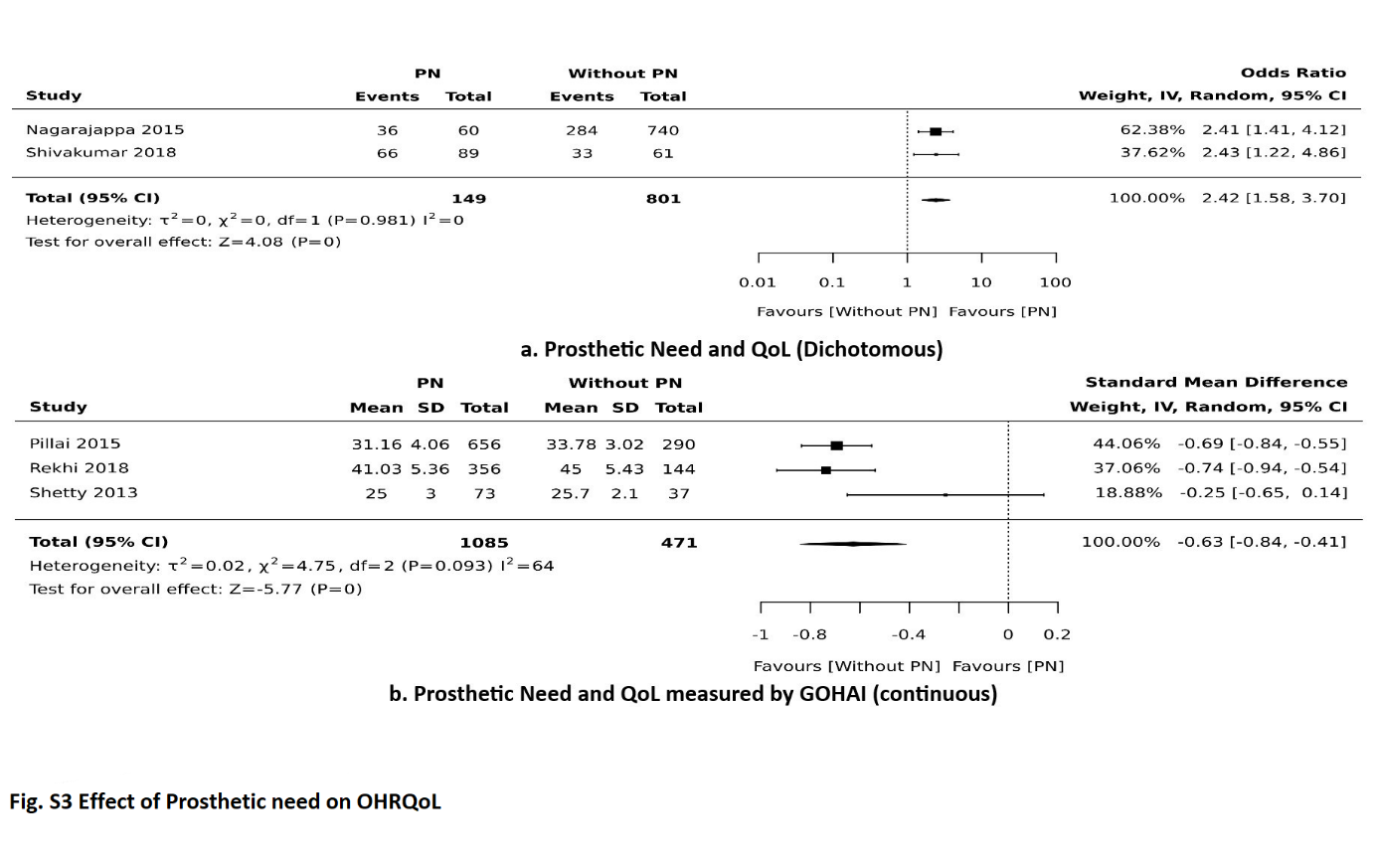


**S6- Effect of prosthetic need on OHRQoL**


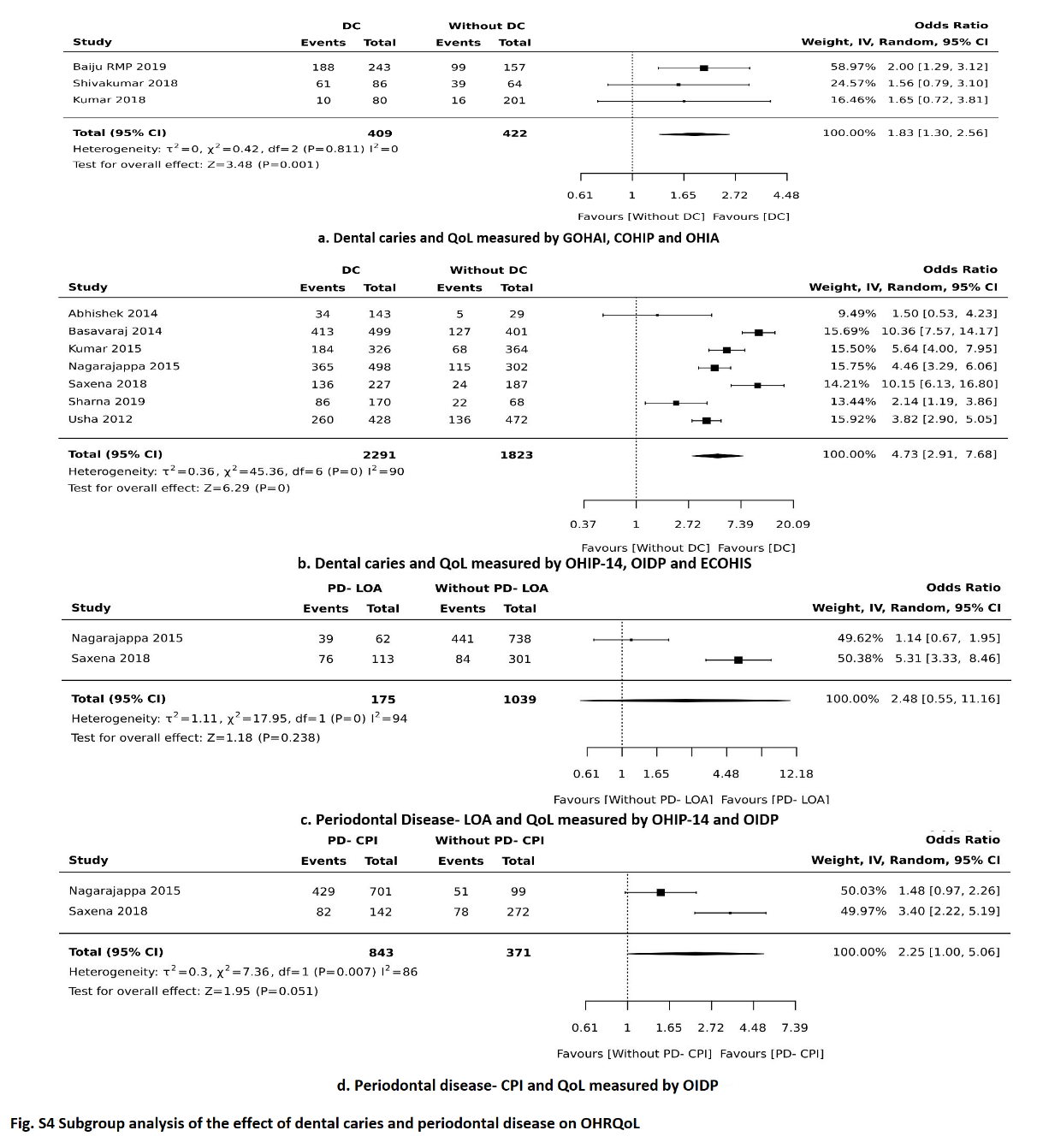


**S7- Subgroup analysis of dental caries and periodontal disease on OHRQoL**
